# Is It Me or You?—How Reactions to Abusive Supervision Are Shaped by Leader Behavior and Follower Perceptions

**DOI:** 10.3389/fpsyg.2018.01309

**Published:** 2018-07-27

**Authors:** Birgit Schyns, Jörg Felfe, Jan Schilling

**Affiliations:** ^1^Department People and Organisations, Center for Leadership and Effective Organizations, Neoma Business School, Reims, France; ^2^Department of Psychology, Helmut-Schmidt University, Hamburg, Germany; ^3^Department of Economics and Social Sciences: Work and Organizational Psychology, University of Applied Administrative Sciences, Hannover, Germany

**Keywords:** abusive supervision, perception, attribution, withdrawal, voice, commitment

## Abstract

There is a growing interest in understanding how follower reactions toward abusive leadership are shaped by followers' perceptions and attributions. Our studies add to the understanding of the process happening between different levels of leaders' abusive behavior (from constructive leadership as control, laissez-faire, mild to strong abusive) and follower reactions. Specifically, we focus on the role of perception of abusive supervision as a mediator and attribution as a moderator of the relationship between leader abusive behavior and follower reactions. Follower reactions are defined in terms of exit, voice, loyalty, and neglect. Two studies using a two point experimental design and vignettes and a cross-sectional field study were conducted. Perception partly mediates the relationship between leader behavior and reactions (Study 1 and 2). Different attributions (intention, control) moderate the relationship between the perception of abusive supervision and reactions in Study 2 and 3. In Study 2, attribution of intentionality of the leader behavior served as a moderator of the relationship between abusive supervision and loyalty, turnover, and voice. Attribution of intentionality reduced the relationship between perception of abusive supervision and reactions. Attribution of intentionality only strengthened negative reactions when milder abusive leadership was perceived. These results were not supported in Study 3. However, in Study 3, attribution to the supervisor' control served as moderator for loyalty and voice. A stronger relationship between the perception of abusive supervision and reactions emerged for high vs. for low attribution to the supervisor. The differences in results between the studies reflect that in Study 1 and 2 abusive behavior was manipulated and in Study 3 the perception of abusive supervision of actual leaders was assessed. Our findings show that avoidance of abusive supervision should be taken seriously and followers' perception and suffering is not only due to subjective judgment but reflects actual differences in behavior. The relationships are stronger in the field study, because, in practice, abusive behaviors might be more ambiguous. The research presented here can help leaders to better understand their own and the followers' role in the perception of and reaction to abusive supervision.

‘*One of the hardest tasks of leadership is understanding that you are not what you are, but what you're perceived to be by others.‘*

Edward L. Flom

The last years have seen a growing interest in the topic of destructive leadership (Schyns and Schilling, 2013; Mackey et al., 2017) with the number of studies investigating the core construct of abusive supervision (Tepper, 2000) having significantly grown over the last decade (Martinko et al., 2013; Tepper et al., 2017). Schyns and Schilling (2013) define destructive leadership as “a process in which over a longer period of time the activities, experiences, and/or relationships of an individual or the members of a group are repeatedly influenced by their supervisor in a way that is perceived as hostile and/or obstructive” (p. 141).

Research into destructive leadership acknowledges that supervisors often do more than simply fail to exhibit constructive behavior toward their followers. Instead, recent meta-analyses demonstrate that strong negative relationships exist between destructive leadership and attitudes toward the supervisor, well-being, job satisfaction, and job-related attitudes (e.g., job engagement), while there are strong positive correlations between destructive leadership and counterproductive work behavior, negative affectivity, and perceived organizational injustice (Schyns and Schilling, 2013; Mackey et al., 2017). This makes the study of abusive supervision a priority. However, it sometimes appears that some followers suffer more under abusive supervisors than others (Brees et al., 2016; Tepper et al., 2017), which has led to an ongoing discussion regarding the distinction between actual abusive supervision and follower perception (Martinko et al., 2013; Tepper et al., 2017). The research presented here aims to add to our understanding of the role of perception in abusive supervision and its negative outcomes. Specifically, we investigate the role of follower perceptions in the relationship between actual behavior and follower behavioral reactions toward abusive supervision. We additionally examine the role of attribution in terms of further explaining different reactions following from abusive perception. In this model, we take into account follower characteristics that are typically associated with perception biases as control variables.

In order to capture reactions, we employ the exit, voice, loyalty, and neglect (EVLN) model (Withey and Cooper, 1989). The EVLN-model was introduced to describe reactions toward dissatisfaction at work. It includes reactions on different levels of severity and is therefore well-suited to examine reactions toward different types of leadership. While there are numerous studies on the outcomes of destructive form of leadership like abusive supervision, we suggest that the EVLN model is particularly helpful to systematize behavioral intentions as reactions toward abusive supervision due to the incremental approach it provides. This is an important first step toward understanding how abusive supervision fosters follower behavior in a more systematic way.

## Introduction

Research into destructive leadership mainly uses follower ratings of their leaders when assessing leader behavior (Tepper et al., 2017). The most used concept, and the one we use here, abusive supervision, is explicitly defined as a perception, namely: “Subordinates' perceptions of the extent to which their supervisors engage in the sustained display of hostile verbal and non-verbal behaviors, excluding physical contact” (Tepper, 2000, p. 178). However, first, using this approach to the measurement of abusive supervision does not provide sufficient information as to whether or not a leader really behaves differently toward their followers or whether or not followers perceive the same behavior in a different way. Second, even if a leader is perceived similarly across followers, their perceptions might not accurately reflect this behavior. Third, we argue that if abusive behavior is not perceived as abusive, the follower will not show negative reactions to these actual behaviors. Naturally, the opposite applies as well that if a behavior is not (or not intended to be) abusive but perceived as such, negative reactions will ensue. Therefore, it is important to study actual leader behavior and how it is related to outcomes via perception, rather than relying on perceptual measurements only. In order to address some of these issues and to be able to draw conclusions about actual behavior and its outcomes, we use an experimental approach, followed by a field study. We argue that even studies using multiple sources such as ratings from different followers of the same leader cannot really disentangle perception from actual behavior. This is in line with Tepper et al. (2017) who make the case that agreement between subordinates does not reflect objectivity, as leaders are likely to vary their behavior toward their followers. Therefore, low intra class correlations (ICC) of leadership ratings may be either indicative of perceptual biases of the followers or of leaders actually behaving differently to different followers.

In addition, previous studies cannot rule out the problem of reverse causality. For example, follower stress is related to the perception of abusive supervision (e.g., Tepper, 2000; Chen and Kao, 2009; for an overview: Schyns and Schilling, 2013). However, there is a possibility that leaders might react negatively to stressed followers so that follower stress influences actual leader behavior, or that the relationship is circular. Similarly, followers' poor performance or negative affectivity may lead to negative reactions by the leader which is perceived as abusive (Wang et al., 2015).

Therefore, to better understand the effects of abusive leadership and the validity of the perception of abusive supervision, it is necessary to investigate abusive supervision in a way which allows us to systematically control actual leadership behavior so that differences in follower perception of and reactions to identical behavior can be attributed to follower characteristics with confidence (see for example Martinko et al., 2012). By using an experimental vignette design (Aguinis and Bradley, 2014), we address these issues. Specifically, we use detailed descriptions of leader behaviors. This way, we can be certain (a) that systematic differences in the perception of different behavior are due to actual behavior and any differences in the perception of (the same) behavior is due to rater effects, and (b) that the relationship between leader behavior and reactions is causal as the leader behavior cannot be influenced by follower behavior or reactions. We are also testing (c) in how far abusive supervision is purely an unspecific perception of any non-positive leadership behavior by including another non-positive leadership style (i.e., laissez-faire). At the same time, by focusing on the EVLN-model relating to outcomes, we investigate effects of leader behavior on outcomes of different severity.

In our studies, we specifically focus on abusive supervision for several reasons. First, abusive supervision is defined as follower perception, thus acknowledging that the same behavior might be perceived differently by followers and can lead to different reactions. Second, we want to add to the discussion regarding the relationship between actual abusive supervision and follower perception/ attribution (Martinko et al., 2013; Tepper et al., 2017) and follower related outcomes. In their comprehensive overview, Martinko et al. (2013, see also Brees et al., 2016) convincingly argue that it is important to distinguish between perceived and actual abusive supervision as we cannot be sure if perceived leader abuse is a valid proxy for actual behavior. In our study, we examine whether or not the perception of abusive supervision mediates the relationship between actual leader behavior and reactions to abusive supervisor behavior, also taking into account rater characteristics commonly found to influence perceptions (Brees et al., 2016).

**Figure 1:**
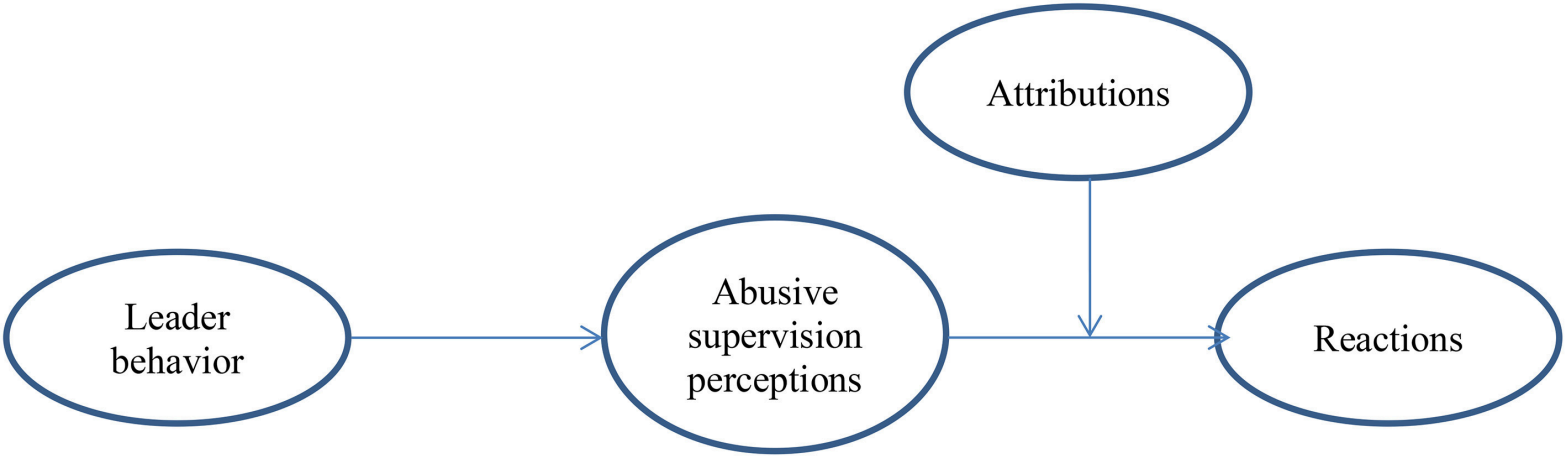
Research model.

We therefore contribute to the existing body of knowledge on abusive supervision by examining the degree to which follower reactions are influenced by actual leader behavior through perceptions. We also add to the question of causality by increasing our knowledge about the most likely direction of relationship between leader behavior and follower reactions. Moreover, by comparing different leadership behaviors (i.e., strong abuse, mild abuse, and laissez-faire leadership), we contribute to the knowledge of the impact of different intensities of negative leadership (Schilling, 2009). In addition, and in line with previous literature suggesting that attribution might be relevant in follower perceptions and reactions to destructive leadership (Martinko et al., 2013), we include attributions to further clarify the process between leader behavior/ follower perceptions and follower reactions. Specifically, we include attribution of intentionality and attribution to the supervisor' control in our study. We argue here that attributions can increase the effect of perceptions of abusive supervision on follower reactions. If abusive leadership is perceived as intentional or as under the control of the supervisor (as opposed to, e.g., results out of incompetence or pressure from outside), followers should react more strongly to the abusive behavior. Figure 1 displays the research model for our studies. In the following we will first draw upon the path between abusive leadership and follower reactions, then address the mediating role of perception and the moderating role of attribution, and finally outline the potential bias of followers' characteristics on perception. Abusive supervision is something that organizations should avoid, and knowing more about the reactions toward abusive supervision can help them do so.

## Abusive leadership and follower reactions

Very often findings of relationships between follower ratings of abusive supervision and outcomes are interpreted as if those follower ratings are a direct and perfect assessment of actual leader behavior (see Martinko et al., 2013, for a critique). However, follower ratings are influenced by rater characteristics such as personality, implicit leadership theories, or affect (for an overview see Hansbrough et al., 2015). Consequently, there is a need to better understand how leader behavior leads to follower reactions via perceptions.

There is, obviously, a myriad of possible reactions to abusive supervisions, and prior research studied a wide variety of outcomes (Schyns and Schilling, 2013). Reactions can happen on different levels (cognitive, emotional, and behavioral) and various coping strategies might come into play when followers are exposed to abusive leadership. We base our choice of reactions on the exit, voice, loyalty, and neglect (EVLN)-Model (Withey and Cooper, 1989), which addresses possible *behavioral* reactions of employees when facing dissatisfactory work situations. That is, we suppose that exit, voice, loyalty, and neglect should all be related to supervisory abuse. This framework is particularly suited to our research as the EVLN framework describes reactions differing in severity. This is helpful for our purposes as we are interested in different levels of leadership which can lead to different levels of reactions.

The most serious reaction would be considering quitting the job (exit). Previous research has shown that abusive leadership increases turnover intentions (Schyns and Schilling, 2013). Although there is a difference between intention and actual behavior, frequently thinking about quitting reduces engagement and enhances the probability of quitting as soon there is an appropriate opportunity.

A more active, however, indirect coping strategy is actively complaining or reducing voice behavior (voice). In comparison to an increasing number of studies focused on aggressive and retaliatory responses of followers (Martinko et al., 2013), actively complaining as a more constructive behavioral alternative did not receive much interest in prior research, so that the current study enters new territory in this respect (Study 1). We also included (in Study 2 and 3), the more traditional assessment of voice (i.e., speaking up), assuming that followers subjected to abusive supervision will show less voice.

While the aforementioned strategies clearly indicate that followers do not accept the perceived behavior, it is also possible that followers lower their attitudes toward their leaders. Indeed, in Schyns and Schilling's (2013) meta-analysis, follower attitudes toward their leaders were the strongest outcome of destructive leadership. Thus, we assess lack of acceptance as loyalty (Study 1). In Study 2, we used a more traditional assessment of loyalty, that is, supervisor commitment, which we assume will decline with abusive supervision.

Finally, neglect is another passive strategy relevant in the study of abusive supervision. Followers may pretend to ignore the situation but in truth brood angrily (Study 1) over the unjust treatment and hope for opportunities to take revenge on the leader or they might passively withdraw from the relationship with the leader (Study 2 and 3).

In the literature around destructive leadership, there is a discussion around how abusive laissez-faire leadership is perceived and how negative it is for follower outcomes. While Schyns and Schilling (2013) explicitly excluded laissez-faire from their meta-analysis arguing that doing nothing is not destructive enough to be a part of destructive leadership, others have argued that laissez-faire is destructive due to its considerable negative consequences (Skogstad et al., 2007, 2014). In line with Schyns and Schilling (2013), we expect that followers react less negatively to non-positive leadership behaviors such as laissez-faire than when exposed to strong abusive leader behavior. At the same time, based on previous research regarding the outcomes of laissez-faire (Skogstad et al., 2007, 2014), we assume that laissez-faire will still instill negative follower reactions. Consequently, with respect to reactions toward the different leadership styles, we assume that reactions are stronger for strong abusive leader behavior than for mild abusive leader behavior, laissez-faire, or constructive leadership. In this respect, the present study offers the opportunity to investigate different forms of negative/ destructive leadership. Experimentally manipulating these types of behavior is especially useful with regard to severe destructive leadership which is difficult to investigate in a field study due to its low frequency.

*Hypothesis 1: Displayed abusive supervision behaviors are positively related to reactions of (a) exit, and (b) neglect, and negatively related to (c) voice and (d) loyalty. The strongest reaction will emerge for strong abusive leader behavior*.

## Perception as mediator between leader behavior and follower reactions

Given our argument that perception is the most important predictor of reactions toward abusive supervision (Martinko et al., 2013), in the sense that if abusive behavior is not perceived as such, there will also not be a reaction toward this behavior, we contend that perception will mediate the relationship between abusive leader behavior and reactions. As Martinko et al. (2013) argued, it is important to distinguish between abusive supervisor behavior and its perception and to investigate its relationship in more depth. As outlined in the introduction, it is important to clarify if the perception of abusive supervision which is typically measured in survey research is a valid proxy for actual behavior. Schyns and Schilling (2013) underline that hostility and obstructiveness of destructive leadership are and can only be subjective evaluations. As Tepper (2000, p. 178) put it “the same individual could view a supervisor's behavior as abusive in one context and as non-abusive in another context, and two subordinates could differ in their evaluations of the same supervisor's behavior.” It seems highly plausible that differences in the perception of whether or not one has been exposed to abusive supervision should be of major importance for subsequent follower reactions. We therefore assume that those evaluations are a necessary prerequisite for any reaction by the follower. Thus we propose the following hypothesis.

*Hypothesis 2: The relationship between displayed abusive supervision behaviors and reactions (a) exit, (b) voice, (c) neglect, and (d) loyalty is mediated by perceptions of abusive supervision*.

## Attribution of abusive supervisor behavior as a moderator in the relationship between perceptions of abusive supervision and follower reactions

We agree with Wang et al. (2015) that it is important to understand the reasons why some individuals react more strongly to perceptions of abusive supervision than others. Attributions have been shown to be important factors in predicting workplace outcomes and reactions (see Harvey et al., 2014, for a recent meta-analysis). We argue that attributions of intentionality and control are particularly relevant here as they will determine the members' perceptions of the leadership behavior (Dasborough and Ashkanasy, 2002).

As Ferris et al. (1995) point out, the way we perceive others' motives for behavior has a distinctive impact on our interpretation and reaction following that behavior. Specifically, we assume that attributing intentionality to perceived abusive behavior will increase the negative effect that abusive supervision has on reactions. While one could argue that abusive behavior is mostly seen as intentional, we think that there can be many reasons to be abusive and that raters will acknowledge these reasons when thinking about intentionality. For example, leaders might push on the pressure they receive from their leader, they might be incompetent, or they might be following norms of their industry or organization. Thus, the extent to which raters attribute intentionality to perceived abusive behaviors can differ. We argue that if raters assume that supervisors use abusive behaviors on purpose rather than maybe due to circumstances, they should react stronger to the abuse. Therefore, we also examine the role of attributions in explaining reactions toward abusive supervision (Study 2 and 3).

Empirically, Lyu et al. (2016) found that attribution style influences the relationship between perceptions of abusive supervision and reactions. They reasoned that “individuals with low hostile attribution bias may attribute their supervisor's hostility as unintentional” (p. 72). More generally, attribution theory (Kelley, 1967; Weiner, 2018) states that causes for behavior can be attributed to person vs. situational reasons. In terms of abusive supervision, we assume that attributions to the person are relevant for reactions. One reason for this assumption is that when the reason for the behavior as situated within the supervisor (rather than the situation) then the behavior is likely to be repeated. Comparing internal, external, and relational attributions for supervisor abuse, Burton et al. (2014) found that attribution to the supervisor was most strongly related to injustice perceptions and related indirectly through injustice to aggression toward the supervisor and lower OCB. Thus, theory and earlier findings argue that attribution to the supervisor is relevant for the reactions of followers to abusive supervision. Therefore, we assume here that the reactions toward abusive supervision will be stronger if followers see the reason for the behavior to be situated in the supervisor. In summary, we propose:

*Hypothesis 3: The mediation of perceptions of abusive supervision of the relationship between displayed abusive supervision behaviors and reactions (a) exit, (b) voice, (c) neglect, and (d) loyalty is moderated by attributed intention/control, such that the relationships will be stronger when the leader's behavior is attributed to the supervisor or his/her intention/control*.

## Follower characteristics as control variables

Based on Martinko et al.'s review (2013) as well as previous research on followers' personality on the perception and acceptance of transformational leadership (Felfe and Schyns, 2010), we argue that follower characteristics influence the perception of abusive supervision. We particularly assume that perceivers who are high in trait negative affectivity, hostile attribution style, anxiety, and irritation (stress) are more likely to perceive leader behavior as abusive. In Mackey et al.'s (2017) meta-analysis, *negative affectivity* was the strongest antecedent of the perception of abusive supervision and therefore we included negative affectivity in our study in order to control for this potential bias. Brees et al. (2016; see also Martinko et al., 2013) highlight the role of *hostile attribution style*, which we also take forward as a follower characteristic. Hoobler and Brass (2006) define hostile attribution style based on Adams and John (1997) as “an extra-punitive mentality where individuals tend to project blame onto others.” Because they interpret others' behaviors in a negative way, individuals high in hostile attribution style are likely to report more abusive supervision than individuals low in hostile attribution style. In addition, we include *anxiety* (as a trait) as a control variable. Mawritz et al. (2014) were able to show that leader anxiety as a state acts as a mediator between leader's hindrance stress (due to exceedingly difficult goals) and perceived abusive supervision. Trait anxiety describes the rather stable tendency of a person to respond to threatening situations with more intense feelings of tension and apprehension and heightened autonomic nervous system activity (Spielberger et al., 1971). We assume that individuals who are more anxious interpret leader behavior more negatively as they are more likely to experience situations as threatening their self-esteem.

We also include *follower stress* in our analyses as, in addition to the effects of personality on the perception of abusive supervision, we expect that followers will be less tolerant and show stronger reactions to abusive supervision when their experienced strain is high. The more followers feel stressed, the more likely they are to perceive abusive supervision (e.g., Tepper, 2000; Chen and Kao, 2009) and react more strongly toward abusive behavior due to their lack of resources to self-regulate (conservation of resource theory, Hobfoll, 1989, and ego-depletion, Baumeister et al., 1998).

## Pre-study: test of vignettes

Similar to previous research in leadership (e.g., Butterfield and Powell, 1981; Rush et al., 1981; Felfe and Schyns, 2006), we used vignettes to describe leader behavior. As the vignettes were used in this study for the first time, we conducted a pre-study to check if they indeed reflect different leadership behavior as we intended them to do. The text of the vignettes is displayed as Supplementary Material. First, we examined in how far the vignettes differ in terms of participants' perceptions of our main concept abusive supervision. We additionally tested our vignettes in terms of how much the described leader is liked by the raters, assuming that the more abusive the leaders are, the less they are liked. We base this assumption on Schyns and Schilling's (2013) finding that abusive supervision is negatively related to follower attitudes toward the leader. In addition, Xu et al. (2012) found a strongly negative relationship between abusive supervision and Leader-Member Exchange, which includes elements of liking. Finally, we investigated to what extent raters would rate the described leaders as more or less leader-like. Again, we assume the more abusive the leader was described, the less leader-like they would be rated. However, also laissez-faire leaders should be rated as not very leader-like as they avoid leading.

### Sample and procedure

We collected a sample of 223 full-time employed participants who currently have a supervisor via an online panel service (Qualtrics)^1^. In order to ensure that participants read the descriptions carefully, we asked them several multiple choice questions as attention checks (“What kind of meeting is it?,” “What is interrupting the meeting?,” “What is the main topic of the meeting?,” and “The employee is a…” man/woman). For each attention check question, we provided three choices (e.g., for question 1, “Face to face business meeting,” “Team meeting,” or “Project kick-off meeting”). Overall, 16 participants did not pass these attention checks, that is, did not answer the questions correctly, leaving a sample of *N* = 207.

The gender was equally distributed (*N* = 104 men, *N* = 103 women). The average age was 25 years old (*SD* = 10 years) and participants had on average worked for their supervisor for 6 months (*SD* = 42 months). We used a between-participants design. The group sizes were as follows: constructive *N* = 47, laissez-faire *N* = 60, mild abusive *N* = 46, and strong abusive *N* = 54.

### Instruments

*Perception of Abusive Supervision* was assessed using Tepper's (2000) 15 item instrument asking the participants to rate the displayed leader's behavior. The scale ranges from 1 = I cannot remember him/her ever using this behavior to 5 = He/she uses this behavior very often. The reliability was α = 0.96. *Liking* was assessed using Engle and Lord's (1997) 5 items instrument. The scale ranges from 1 = strongly disagree to 5 = strongly agree. The reliability was α = 0.96. *Generalized Leadership Impression* (GLI; Cronshaw and Lord's (1987) was used to assess leader-likeness. The instrument consists of 5 items. The scale ranges from 1 to 5 with different anchors. A high value indicates a high leadership impression. The reliability was α = 0.91.

### Results and discussion

Table 1 displays the mean values for the four vignettes regarding abusive supervision, liking, and generalized leadership impression. In terms of the perception of abusive supervision, the differences were as expected with constructive leadership being perceived as the least abusive. All mean differences were significant (*p* < 0.01), except for the difference between mild and strong abusive supervision. The ranking of the means was in the expected direction, with constructive leader behavior having the lowest mean and strong abusive leader behavior showing the highest mean.

**Table 1:** Mean values for abusive supervision, liking, and generalized leadership impression for the four vignettes (pre-study).

**Constructive**	**Laissez-faire**	**Mild abusive**	**Strong abusive**
**PRE-STUDY**			
**Abusive Supervision**			
1.80 (0.76)_c_	2.68 (0.83)_b_	3.24 (0.90)_a_	3.54 (0.97)_a_
**Liking**			
2.85 (0.94)_a_	1.65 (0.95)_b_	1.43 (0.66)_b, c_	1.16 (0.41)_c_
**GLI**			
3.38 (0.68)_a_	1.95 (0.87)_b_	2.00 (0.84)_b_	1.67 (0.82)_b_
**STUDY 1**			
**Abusive Supervision**			
1.56 (0.67)_d_	2.72 (0.87)_c_	3.15 (0.87)_b_	3.69 (0.81)_a_
**Liking**			
3.46 (0.81)_a_	1.62 (0.70)_b_	1.61 (0.75)_b_	1.25 (0.54)_c_
**GLI**			
3.51 (0.75)_a_	1.90 (0.66)_b_	1.89 (0.70)_b_	1.53 (48)_c_

*Standard deviations in brackets. Pre-study—Abusive Supervision F_(3, 203)_ = 37.83, p < 0.001; Liking F_(3, 203)_ = 44.87, p < 0.001; GLI F_(3, 203)_ = 43.74, p < 0.001; Study 1—Abusive Supervision F_(3, 306)_ = 93.22, p < 0.001; Liking F_(3, 306)_ = 147.62, p < 0.001; GLI F_(3, 306)_ = 134.58, p < 0.001; Going across rows, mean values with different letters are significantly different from each other (minimum p < 0.05). “a” stands for the highest mean value, b for the second highest, etc. (post-hoc Bonferoni test)*.

In terms of liking, there was also a non-significant difference between mild and strong abusive supervision. Here, also the difference between laissez-faire and mild abusive was not significant. The ranking of the means was in the expected direction, with constructive leader behavior having the highest mean and strong abusive leader behavior showing the lowest mean. For the impression of the leaders as leader-like, constructive was significantly different from all other styles. That is, only the leader showing constructive behavior was considered leader-like. Apart from the leadership impression, all outcomes were in the expected ranking order from constructive via laissez-faire and mild abusive to strong abusive. We assume that leaders who show laissez-faire are equally considered unlike leaders as abusive leaders. With a larger sample size, the results would have most likely become significant. Also, some values were already low, leading to a floor effect and thus a lower likelihood of finding significant differences. However, we take the correct ranking of the vignettes in terms of our criterion variables as an indicator that our vignettes are a useful means to represent different leadership styles^2^. Hence, based on these results, we assume that we can use the different vignettes as representing varying degrees of abusive behavior (from non-abusive: constructive to strong abusive).

## Study 1

### Design and procedure

We used an online provider (Qualtrics) to collect the sample. The study reported here was part of a larger study. In order to separate measurements, we asked participants to fill out two questionnaires. At time 1, we asked them to indicate stable characteristics, that is, negative affectivity, and hostile attribution style. At time 2 (about a week later), we asked participants about their stress (irritation). We also gave them the descriptions of the leaders to read (between participants design: constructive *N* = 71, laissez-faire *N* = 76, mild abusive *N* = 82, and strong abusive *N* = 81). Only participants who answered both questionnaires and correctly responded to the attention check questions (see pre-study) were maintained in the sample to test the hypotheses. Ninety participants did not pass the attention checks. After reading the vignette, participants rated the described leader on the abusive supervision scale and indicated their anticipated reactions to such a leadership behavior.

### Participants

The sample consisted of 310 full-time working employees, 162 men, 148 women, with an average of age 45 (SD = 11). The average work experience was 22.6 years (SD = 12.13). We did not ask about managerial experience as we were interested in our participants' views as followers.

### Instruments

#### Follower characteristics as control variables: negative affectivity

Follower characteristics as control variables: Negative affectivity (T1) was assessed using 10 items relating to negative affectivity of the PANAS (Watson et al., 1988). The reliability was α = 0.91 (1 = very slightly / not at all to 5 = extremely). *Hostile attribution style* (T1) was assessed using an instrument by Hoobler and Brass (2006). The reliability for six items was α = 0.76 (1 = strongly disagree to 5 = strongly agree). *Stress* was assessed (at T2) using the irritation instrument by Mohr et al. (2006). The reliability for the 8 items was α = 0.90 (1 = strongly disagree to 7 = strongly agree).

#### Leader behavior

Leader behavior was displayed as described in the pre-study.

#### Perception of abusive supervision

We used Tepper's (2000) 15-item instrument to assess the perception of abusive supervision of the displayed leader behavior at T2. The reliability was α = 0.96.

#### Reactions to abusive supervision

We created an instrument to assess the reactions to abusive supervision. We were particularly interested in the acceptance of abusive supervision (loyalty), complaining about the supervisor (voice), in how far people wanted to quit (exit) after being exposed to an abusive supervisory style, and in how far they anticipated showing an anger reaction (neglect) to abusive supervision (based on the EVLN-model). We used two items to assess each of those reactions. For the first item of each reaction, the response was 1 = certainly not to 6 = certainly; for the second item of each reaction, it was 1 = very poor way of dealing with it, 4 = very appropriate way of dealing with it. For *Quit*, we used: “I think about leaving and changing my job.” *Anger* was assessed with “I swallow my anger and wait for a chance to pay my supervisor back by some means or other.” For *complain*, we used “I'm going to complain to a higher authority, because this is not the way you should treat people.” Finally, *accept* was assessed with “I will do as I am told, because my supervisor is right and it is my fault.” An exploratory factor analysis supported the four factor structure.

### Results

We examined in how far the vignettes differed in terms of perceived abusive supervision and reactions using ANOVAs (see Table 2). The results show that the perception of abusive supervision differed between the vignettes in the expected direction. With regard to H1, for the reactions “quitting” and “anger” the differences between laissez-faire, mild abusive, and strong abusive supervision were not significant but all are significantly different from constructive leadership in the expected direction, namely that for constructive leadership these reactions were significantly lower compared to all other conditions. For the reaction “complaining,” all differences were significant apart from the differences between laissez-faire and mild abusive supervision. For “acceptance” differences between laissez-faire, mild abusive, and strong abusive supervision were not significant. Here, the differences between constructive and laissez-faire as well as mild and strong abusive were significant. The difference between mild abuse and laissez-faire was significant. The difference between mild and strong abusive was significant at *p* < .05. These results lend support to Hypothesis 1. Intercorrelations are shown in Table 3.

**Table 2:** Mean values for reactions for the four vignettes (Study 1).

**Constructive**	**Laissez-faire**	**Mild abusive**	**Strong abusive**
**Quitting**			
2.01 (0.92)_b_	3.11 (0.88)_a_	3.30 (0.89)_a_	3.31 (0.92)_a_
**Anger**			
1.82 (0.96)_b_	2.39 (0.96)_a_	2.41 (97)_a_	2.30 (0.97)_a_
**Complain**			
1.71 (0.84)_c_	3.06 (0.88)_b_	3.41 (0.90)_b_	3.99 (0.90)_a_
**Accept**			
3.37 (0.97)_a_	2.20 (0.88)_b_	2.33 (0.85)_b_	2.06 (0.90)_b_

*Standard deviations in brackets. Quitting F_(3, 306)_ = 34.74, p < 0.001; Anger F_(3, 306)_ = 5.92, p < 0.005; Complain F_(3, 306)_ = 89.28, p < 0.001; Accept F_(3, 306)_ = 32.47, p < 0.001; Going across rows, mean values with different letters are significantly different from each other (minimum p < 0.05). “a” stands for the highest mean value, “b” for the second highest, etc. (post-hoc Bonferoni test)*.

**Table 3:** Intercorrelations for abusive supervision, control variables, and reactions (Study 1).

	**M**	**SD**	**1**	**2**	**3**	**4**	**5**	**6**	**7**
Perc. abusive supervision (T2)	2.82	1.11							
Negative Affectivity (T1)	1.78	0.69	0.06						
Hostile attribution style (T1)	2.74	0.65	0.09	0.25^**^					
Irritation (T2)	3.41	1.31	0.05	0.57^**^	0.20^**^				
Reaction: Quit	2.96	1.04	0.50^**^	0.10	0.03	0.14^*^			
Reaction: Anger	2.24	0.99	0.18^**^	0.12^*^	0.27^**^	0.13^*^	0.30^**^		
Reaction: Complain	3.09	1.20	0.60^**^	−0.02	0.04	−0.11^*^	0.43^**^	0.08	
Reaction: Accept	2.47	1.03	−0.40^**^	0.16^**^	0.03	0.15^**^	−0.30^**^	−0.04	−0.57^**^

* p < 0.05 (2-tailed)

** *p < 0.01 (2-tailed)*.

**Figure 2:**
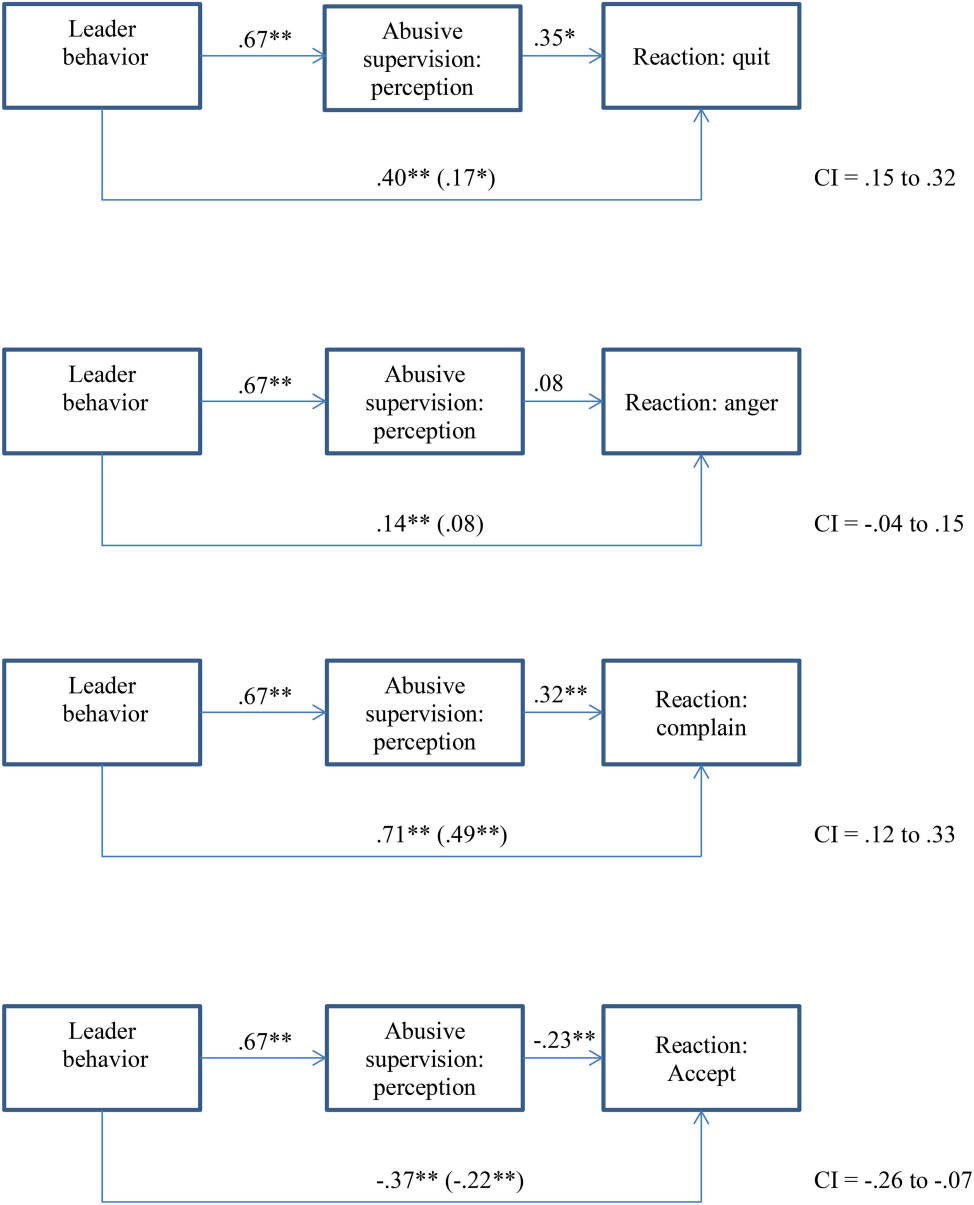
Mediation of perception of abusive supervision on the relationship between abusive supervision behavior and reactions (including controls; Study 1). *p < 0.05 (2-tailed), **p < 0.01 (2-tailed).

In order to test H2, we conducted mediation analyses using the PROCESS macro (Preacher and Hayes, 2008). Based on our pre-test of the vignettes, we used the vignettes as a continuous predictor, representing a continuum from not abusive at all (constructive) to strong abusive behavior. We used negative affectivity and hostile attribution style to control for the influence of personality and stress to control for resource depletion that might also affect the perception of abusive leadership^3^. Apart from the mediation of perception of abusive supervision on the relationship between abusive supervision behavior and anger reactions, all other mediation effects were supported, lending partial support to our H2 (see Figure 2). With respect to the control variables, we did not find any relationships with perception. The control variables were, however, related to some of the reactions: for quitting irritation was significant, for anger, irritation and hostile attribution style, and for accepting, negative affectivity.

### Discussion study 1

The aim of our first study was to examine in how far different leader behaviors are related to different follower reactions, mediated by the perception of abusive supervision. As expected, we found the strongest effect of strong abusive behaviors on reactions. There has been some discussion around the negative effects of laissez-faire leadership (Skogstad et al., 2014) and whether or not laissez-faire might be even worse than abusive supervision. Our results do not support this consideration. Although mild abuse and laissez-faire did not differ on all outcome variables, they did differ in terms of the perception of how abusive the described leader is.

We also found that perception of abusive supervision partially mediates the relationship between behavior and most reactions. We could show here that in the process of abusive supervision, perception is relevant and can influence the strength of the reaction to leader behavior. At the same time, it is clear that leader behavior remains a strong influence on reactions. Perception appears to be a valid proxy of actual behavior; however perception does not capture the entire effect of behavior. Moreover, some follower characteristics (irritation, hostile attribution style, and negative affectivity) were related followers' reaction but not to perception. This also means that follower reactions cannot be simply put down to sensitivities of the follower but that leader behavior is a crucial factor in reactions toward negative leader behavior.

## Study 2

### Overview

In Study 2, we extended Study 1 and addressed limitations. First, in Study 1, reactions in terms of EVLN were measured with a self-constructed questionnaire. In Study 2, we use more established questionnaires to assess reactions. In addition, we also asked participants to rate their attribution of intentionality of the described leader behavior. We assumed that attribution of intentionality will moderate the relationship between perception of leadership and reactions. In line with our theoretical assumptions regarding influences of follower characteristics on ratings of abusive supervision, we added trait anxiety to control for the influence of follower characteristics. We assume that anxious individuals interpret leader behavior more negatively and react accordingly.

### Design and procedure

We again used an online provider (respondi) to collect the sample. As in Study 1, we asked participants to fill out two questionnaires. At time 1, we asked them to indicate stable characteristics, that is, negative affectivity, hostile attribution style, and trait anxiety. At time 2 (about 2 days later), we first asked participants about their experienced stress (irritation). We then gave them the descriptions of the leaders to read (between participants design: constructive *N* = 60, laissez-faire *N* = 57, mild abusive *N* = 56, and strong abusive *N* = 61). Participants rated the described leader on the abusive supervision scale (perceived leader behavior), answered four items regarding the attribution of the described behavior (intentional) and finally, indicated their anticipated reactions to such a leadership behavior.

### Participants

As before, we only included participants who filled in the questionnaires at both times and passed the attention checks (see pre-study for the questions). Sixty-three failed our attention checks. The final sample consisted of *N* = 234 participants. Of those 141 were men, 93 women. Most participants were between 45 and 54 years old (30%).

### Measures

#### Follower characteristics as control variables

Negative affectivity (T1, α = 0.90), hostile attribution style (T1, α = 0.75), and stress (T2, α = 0.92) were assessed using the same instruments as in Study 1. *Anxiety* (T1) was assessed with four items. One item stemmed from the neuroticism subscale of the Big Six (Ashton et al., 2004) and three were taken from the BFI (John and Srivastava, 1999) (1 = strongly disagree to 5 = strongly agree; α = 0.89).

#### Leader behavior (T2)

Leader behavior (T2) was displayed with the vignettes described in the pre-study and Study 1.

#### Perception of abusive supervision (T2)

We again used Tepper's (2000) abusive supervision scale (α = 0.97).

#### Attribution of intentionality (T2)

We used one item to assess attribution of the described leader behavior. Participants were asked to indicate on a scale from 1 (strongly disagree) to 7 (strongly agree) in how far they agree with the following statement. “The leader behavior: …is clearly intentional on part of the supervisor.”

#### Reactions to abusive supervision (T2)

As in Study 1, we based our assessment of reactions to leadership on the EVLN-model (Withey and Cooper, 1989). We asked people to indicate “If you were in this situation, please rate what would be most likely for you to do or think in this situation” on different instruments. We assessed *Exit* using three items relating to turnover intention (Hom et al., 1984; Mitchell et al., 2001). A sample item reads “Would you intend to leave the organization in the next 12 months?” (α = 0.96; 1 = certainly not to 6 = certainly). *Voice* was assessed using Liang et al. (2012) ten item measure (α = 0.93; 1 = never to 5 = frequently). In line with Liang et al. (2012), we differentiated between promotive (α = 0.97) and prohibitive voice (α = 0.88). For *loyalty*, we used the 5 items supervisor commitment scale (α = 0.96; 1 = never to 5 = frequently; Felfe et al., 2006). For *neglect*, we used a three items measurement of withdrawal (α = 0.95; “I would withdraw from this supervisor”; 1 = never to 5 = frequently) based on Aquino et al. (2006).

### Results

As in Study 1, we calculated ANOVAs to examine the differences between the vignettes concerning abusive supervision perceptions as well as the reactions. Table 4 shows the results. All mean values ranked in the expected order. With respect to the reactions (H1), again the ranking of the mean values was as expected, though some differences between the means were not significant. Overall Fs were significant apart from prohibitive voice. This lends support to H1.

**Table 4:** ANOVA results for the differences between the vignettes on perceptions and reactions (Study 2).

**Constructive**	**Laissez-faire**	**Mild Abuse**	**Strong Abuse**
**MEAN VALUES**			
**Abusive supervision**			
1.79 (0.78)_c_	2.53 (1.09)_b_	2.98 (0.92)_a, b_	3.12 (1.22)_a_
**Turnover intention**			
3.17 (1.42)_c_	4.16 (1.22)_b_	4.64 (1.24)_b_	5.27 (1.02)_a_
**Supervisor commitment**			
2.42 (1.13)_a_	1.62 (0.91)_b_	1.39 (0.68)_b_	1.27 (0.59)_b_
**Withdrawal**			
2.64 (1.34)_c_	3.52 (1.15)_b_	3.90 (1.05)_b_	4.49 (1.08)_a_
**Prohibitive voice**			
3.13 (0.81)_a_	3.05 (0.86)_a_	3.00 (0.92)_a_	2.79 (1.13)_a_
**Promotive voice**			
3.50 (0.95)_a_	3.26 (1.01)_a, b_	2.92 (1.14)_b_	2.20 (1.08)_c_

*Abusive supervision F_(3, 230)_ = 20.99, p < 0.001; turnover intention F_(3, 230)_ = 31.24, p < 0.001; supervisor commitment F_(3, 230)_ = 22.03, p < 0.001; withdrawal F_(3, 230)_ = 30.44, p < 0.001; prohibitive voice F_(3, 230)_ = 1.39, n.s., and promotive voice F_(3, 230)_ = 17.75, p < 0.001. Going across columns, mean values with different letters are significantly different from each other (minimum p < 0.05). “a” stands for the highest mean value, “b” for the second highest, etc*.

Prior to conducting our mediation and moderation analyses, we correlated the rater characteristics assessed at T1 (negative affectivity, anxiety, and hostile attribution style) as well as stress assessed at T2 with abusive supervision assessed at T2 in order to examine rater biases in abusive supervision ratings independent of the leader behavior described to the participants. As can be seen in Table 5, the correlations between the perception of abusive supervision and negative affectivity, anxiety and irritation were positive and significant. There was no significant correlation between hostile attribution style and abusive supervision. We also conducted a hierarchical regression analysis with all control variables predicting abusive supervision (first negative affectivity, second hostile attribution style, third anxiety, and last irritation). While the *R*^2^ change was significant for negative affectivity, none of the controls was significant when all were entered into the regression. Therefore, only negative affectivity was included as a control in our moderated mediation analyses.

**Table 5:** Means, standard deviations and, intercorrelations (Study 2).

	**M**	**SD**	**1**	**2**	**3**	**4**	**5**	**6**	**7**	**8**	**9**	**10**
Perc. abusive supervision	2.60	1.14										
Negative affectivity T1	1.87	0.70	0.20^**^									
Hostile attribution style T1	2.80	0.73	0.04	0.27^**^								
Anxiety	3.08	1.05	0.18^**^	0.59^**^	0.16^**^							
Irritation	3.58	1.37	0.23^**^	0.55^**^	0.20^**^	0.62^**^						
Turnover intention	4.31	1.45	0.59^**^	0.16^*^	0.04	0.13^**^	0.20^**^					
Supervisor Commitment	1.68	0.96	−0.50^**^	−0.11	0.01	−0.09	−0.17^*^	−0.73^**^				
Withdrawal	3.64	1.28	0.51^**^	0.25^**^	0.05	0.22^**^	0.20^**^	0.74^**^	−0.69^**^			
Prohibitive Voice	2.99	0.94	−0.09	−0.23^**^	−0.05	−0.21^**^	−0.15^*^	−0.20^**^	0.24^**^	−0.30^**^		
Promotive Voice	2.96	1.15	−0.34^**^	−0.21^**^	−0.09	−0.17^**^	−0.15^*^	−0.45^**^	0.46^**^	−0.57^**^	0.56^**^	
Intentionality	5.43	1.46	0.03	−0.03	−0.03	0.06	−0.00	0.23^**^	−0.22^**^	0.25^**^	−0.01	0.16^*^

* p < 0.05 (2-tailed)

** *p < 0.01 (2-tailed)*.

While there was no significant correlation between abusive supervision and attributed intentionality (*r* = −.03), intentionality was positively related to all outcomes apart from prohibitive voice. Perception of abusive supervision was related to all outcomes as expected, again apart from prohibitive voice.

**Figure 3:**
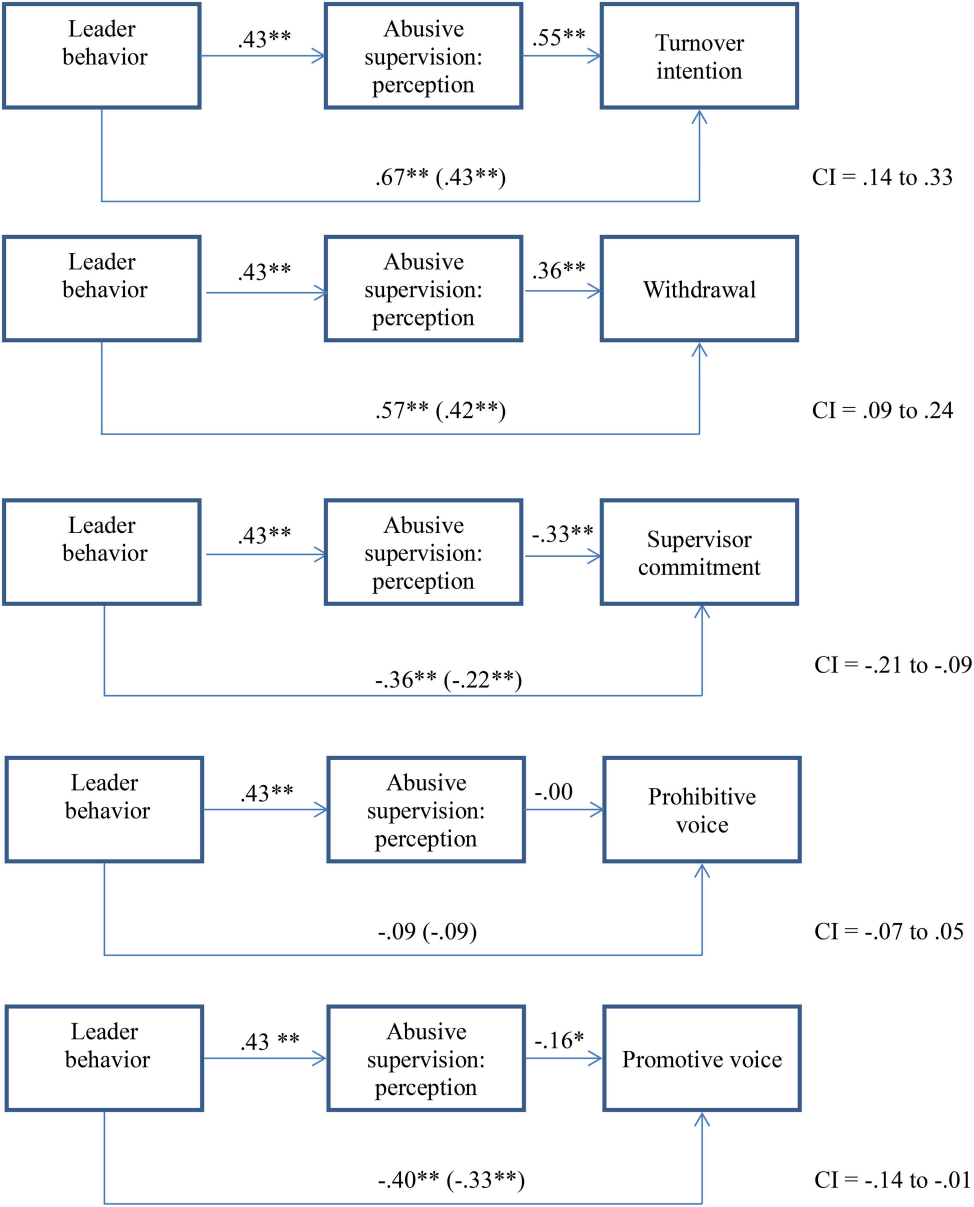
Mediation of perception on the relationship between behavior and reactions (including negative affectivity as control; Study 2). *p < 0.05 (2-tailed), **p < 0.01 (2-tailed).

In the following step, we conducted mediation analyses using Preacher and Hayes (2008) bootstrapping method to examine if we can replicate the results of Study 1 with the improved assessments for the outcome variables. As before, we used the vignettes as a continuous predictor. In terms of H 2, Figure 3 depicts that all indirect effects are significant apart from prohibitive voice. The direct effects were substantially reduced when perception of abusive supervision was included as a mediator, again apart from prohibitive voice. In terms of the control variables, negative affectivity was significant for all but supervisor commitment at least on the *p* < 0.05 level. Figure 3 shows the mediation results with control variable (negative affectivity). This lends support to our H2.

Finally, we tested a moderated mediation model (H3), in which attribution of intention moderates the relationship between the mediator perception of abusive supervision and the reaction variables. As intention in our model is only relevant when it relates to negative behavior, we only used the two groups that read the negative leadership behavior description, that is, mild and strong abusive leadership. This reduced our sample size to *N* = 117.

**Figure 4:**
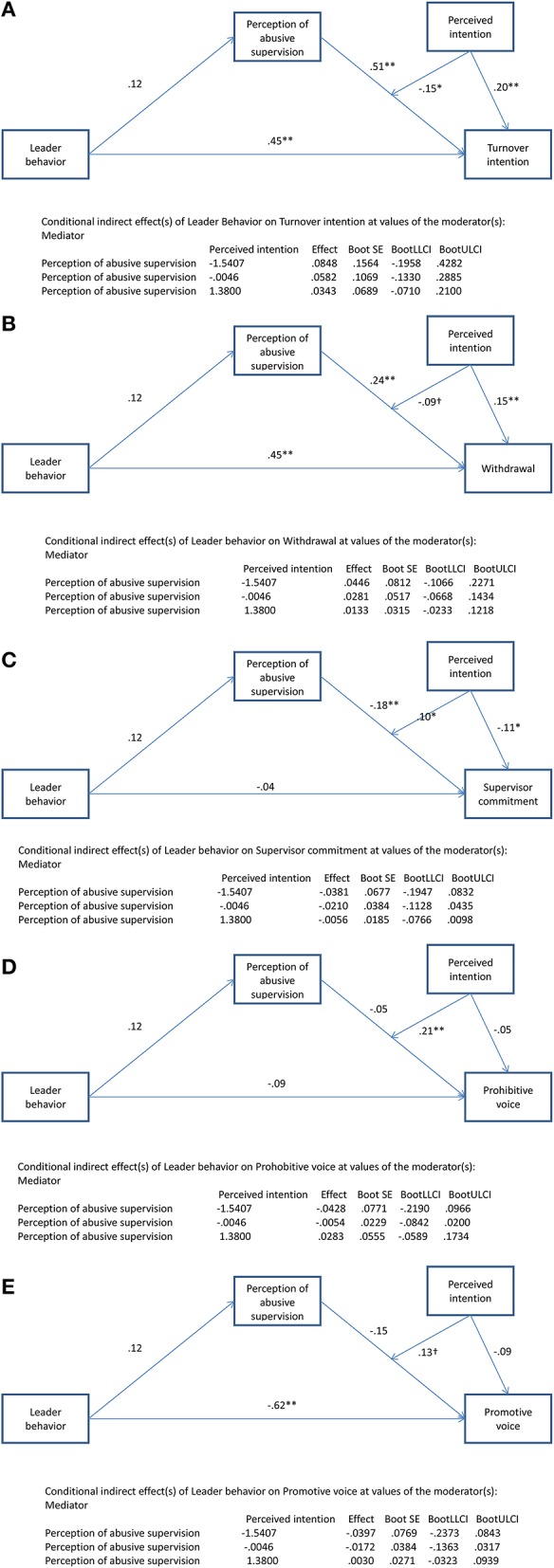
**(A–E)** Moderated mediation of intention and perception of abusive supervision on the relationship between abusive supervision behavior and reactions (including negative affectivity as control, only abusive vignettes) Study 2. ^†^*p* < 0.10 (2-tailed), *p < 0.05 (2-tailed), **p < 0.01 (2-tailed).

For exit, the direct effect of the vignettes was .45 (*p* < 0.05). The interaction between perception of abusive supervision and intention was significant (−0.15, *p* < 0.05). For withdrawal, the direct effect of the vignettes was significant at .45 (*p* < 0.01). The interaction between perception of abusive supervision and intention was significant (−0.09, *p* < 0.10). For supervisor commitment, the direct effect of the vignettes was −0.04 (n.s.). The interaction between perception of abusive supervision and intention was significant (0.10, *p* < 0.05). For prohibitive voice, the direct effect of the vignettes was −.09 (n.s.). The interaction between perception of abusive supervision and intention was significant (0.21, *p* < 0.01). For promotive voice, the direct effect of the vignettes was significant (−0.62, *p* < 0.01). The interaction between perception of abusive supervision and intention was significant (0.13, *p* < 0.10). In summary, apart from withdrawal and promotive voice (both interactions *p* < 0.10) as independent variables, all moderated mediation effects were significant at least at *p* < 0.05 (see Figures 4A–E).

**Figure 5:**
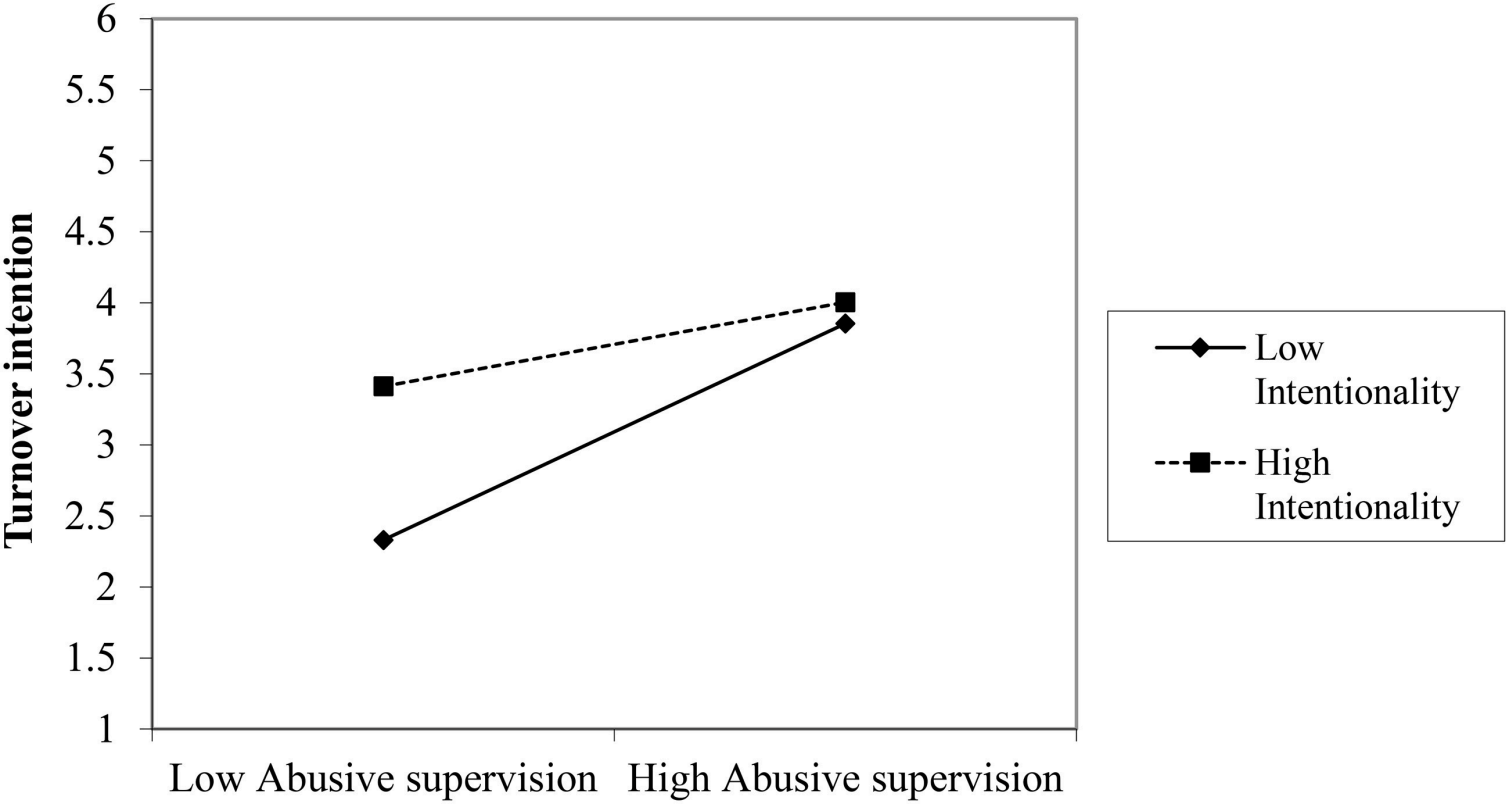
Interaction between abusive supervision and attribution to the supervisor on turnover intention (Study 2).

**Figure 6:**
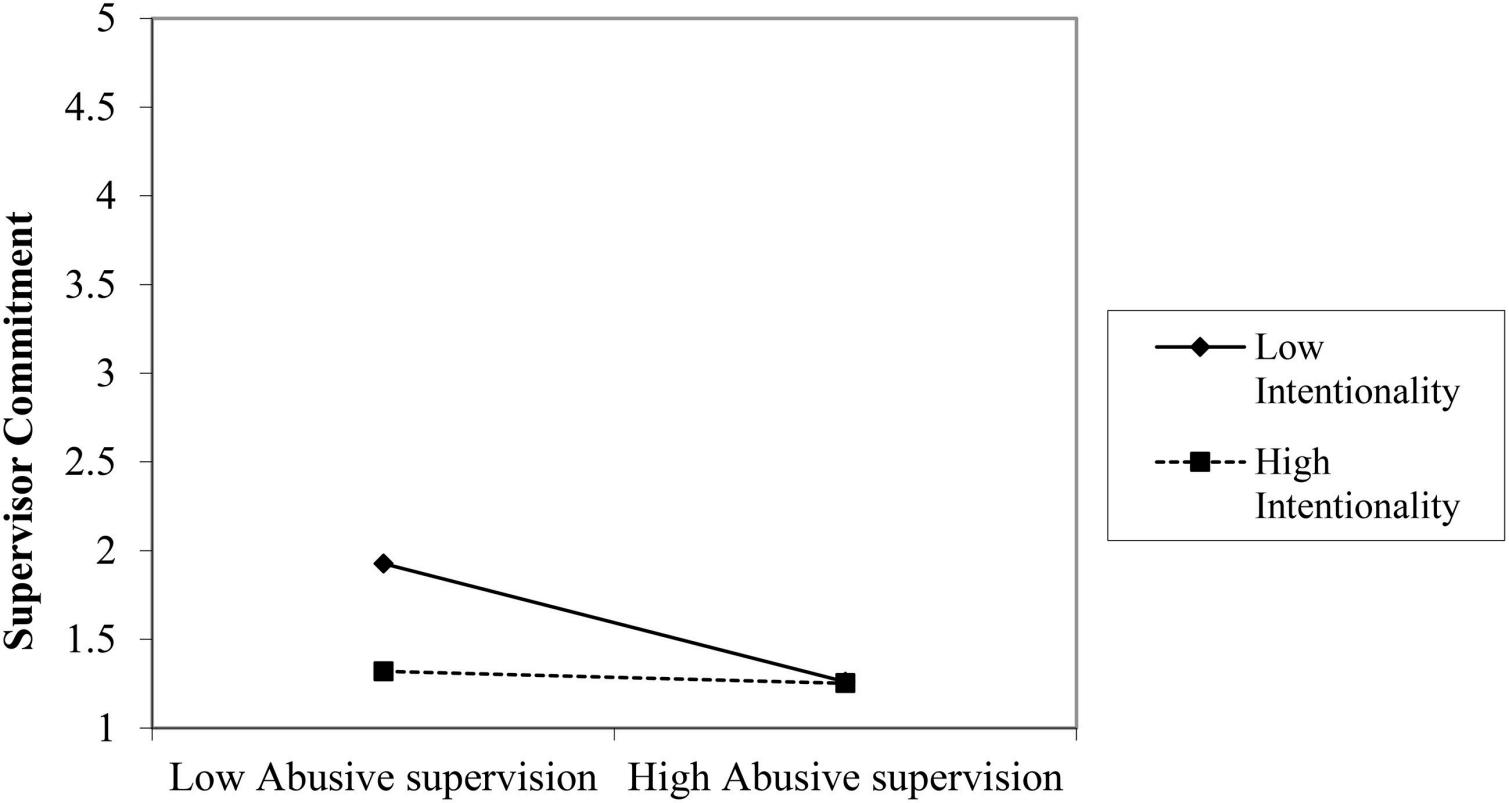
Interaction between abusive supervision and attribution to the supervisor on supervisor commitment (Study 2).

**Figure 7:**
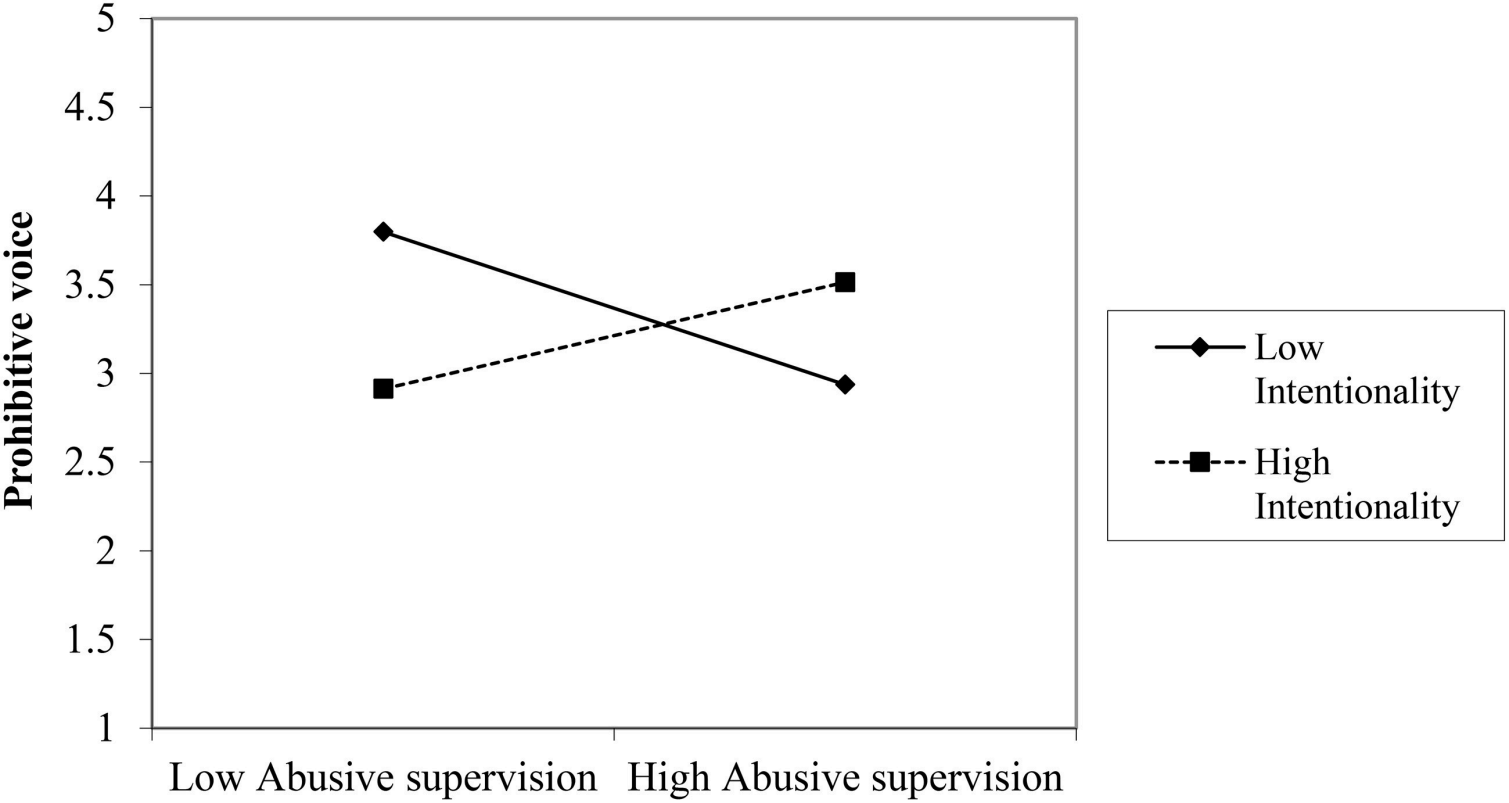
Interaction between abusive supervision and attribution to the supervisor on prohibitive voice (Study 2).

We plotted the interactions for turnover intention, supervisor commitment, prohibitive voice (see Figures 5–7)^4^. For high attribution of intentionality, the relationship between abusive supervision and turnover intention was less negative than for low intentionality, but overall, turnover intention was on a higher level. The interaction thus shows that at high abusive supervision, turnover intention is high, independent of intentionality and that intentionality is relevant at lower levels of abusive supervision (note that the data used here only reflects the two abusive vignettes). Though not plotted here, the pattern was the same for withdrawal. For high attribution of intentionality, the relationship between abusive supervision and supervisor commitment was near zero, whereas for low attribution of intentionality, the relationship between abusive supervision and supervisor commitment was negative. Again at high levels of abusive supervision, attribution of intentionality makes little difference to the reaction of the participant in terms of supervisor commitment. Whereas at low levels of abusive supervision, attribution was relevant for supervisor commitment, this was not the case at high levels of abusive supervision. Looking at prohibitive voice as an outcome, the relationship between abusive supervision and prohibitive voice changed from positive (for high attribution of intentionality) to negative (for low attribution of intentionality). Thus, voice reduced under low intentionality with abusive supervision and increased under high intentionality with abusive supervision. Though not plotted here, the pattern was the same for promotive voice. This lends support to H3.

### Discussion study 2

In Study 2, we replicated (most of) the results of Study 1 in terms of the mediation effect of the perception of abusive supervision on the relationship between abusive leader behaviors and reactions, again in an experimental design with improved measures for reactions, including a differentiation between prohibitive and promotive voice. We also added attribution of intentionality as a moderator of this mediation relationship and found that attribution of intentionality is less relevant for turnover and supervisor commitment when high abusive supervision is perceived than when abuse is perceived as lower. In other words, with high abusive supervision, there is a high level of turnover intention and a low level of commitment while attribution of intention does not make a difference. In terms of voice, the relationship between abusive supervision and voice turned positive for high attribution of intentionality. We wonder if this is a limitation of our design, where participants were asked to indicate how they *would* react to an abusive supervisor. Participants might think that they would use more proactive behaviors to counteract abusive (as those behaviors are not necessarily directed directly to the supervisor). However, this result is unlikely to replicate in our field Study 3 where we ask for actual reactions. Here, voice might go down when intentionality is attributed to abusive behavior due to fear of retaliation or being subjected to more abuse when the follower makes him/herself more visible through the use of voice.

So far, all our studies used experimental designs to control for leader behavior and allow for a better control of perception effects as well as drawing conclusions about causality of the effects. In Study 3, we examined if the results for attribution replicate in a field study. We also assume here that rater characteristics will have a stronger impact on the perception of abusive supervision as actual behavior is more ambiguous in the field and thus we expect more rater bias than in our experimental studies.

## Study 3

### Overview

In Study 3, we wanted to examine our model using the perception of abusive supervision of actual leaders in a cross-sectional field study. While this design does not allow drawing conclusions about causality, it is aimed to add more external validity to our experimental results. At the same time, we expect that actual leader behavior is more ambiguous and that, consequently, we will find a stronger effect of the control variables (follower characteristics; specifically, negative affectivity, hostile attribution style, trait anxiety, and irritation) on the perception of abusive supervision in the field study, comparable to previous studies (see Martinko et al., 2013, for an overview). As mentioned in the introduction, we also assume that we will find overall low levels of perceived abusive supervision as especially strong abuse is seldom. Hence, the field study complements our experiments by adding ecological validity to our results.

In order to further explore the moderating effect of attribution, we added a second measurement of attribution, namely attribution to the supervisor's control in addition to our intentionality assessment. Similarly to attribution to intentionality, we assumed that attribution to the supervisor's control would increase the strength of the relationship between perceptions of abusive supervision and reactions.

### Design and procedure

We again used an online provider (respondi) to collect the sample. However, this time, we did not ask the participants to read vignettes and evaluate a described leader but to evaluate their own leader. First, we asked the participants to indicate a few stable characteristics, namely, trait negative affectivity, hostile attribution style, irritation, and anxiety. Second, participants rated their leader's abusive supervision and their attributions for this behavior. They also indicated their intention to quit, commitment to their supervisor, voice behavior, and withdrawal from the relationship with their supervisor.

### Participants

We used the same inclusion criteria as in Study 2 to keep the samples comparable. Specifically, participants were employed, between 18 and 65 years old, and had 3 or more months of work experience. The sample size after applying quality checks (e.g., lack of standard deviations, *N* = 56 did not pass our quality checks) was *N* = 313. The majority (55.2%) was between 35 and 54 years old. Of the participants, 182 were male (58.1%) and 131 were female (41.9%). The majority of the participants had 10 or more years of work experience (75.4%). About a third (31.6%) had A-levels, 28.4% had a graduate degree, 25.9% GCSEs and 14.1% had a postgraduate degree. The participants came from various industries such as health care, government, or retail. On average, they worked with their supervisor since 4 years (SD = 4.54).

### Measures

Unless otherwise stated, the scale ranges are the same as in Study 2. The instruments for *Negative affectivity* (α = 0.92), *Hostile attribution style* (α = 0.80), *Trait anxiety* (α = 0.88) and *Irritation* (1 = strongly disagree to 5 = strongly agree; α = 0.89) were the same as in Study 2. We also used the same instrument for *Perception of abusive supervision* (α = 0.96). However, the items now referred to the participants' actual leader.

We used two measurements of *Attribution*. First, we used the one item measurement used in Study 2 referring to intentionality but now relating to their actual supervisor. Second, we adapted a three-item instrument by McAuley et al. (1992) to assess in how the participants attributed to their supervisor on a semantic differential ranging from 1 to 9. A sample questions reads “Is it a behavior… Over which he/she has control / over which he/she has no control.” The reliability was α = 0.92.

*Reactions to abusive supervision* were assessed with the same instruments as in Study 2 but now relating to their actual experience: *Exit/Turnover intention* (α = 0.96), v*oice* (overall α = 0.92, promotive α = 0.96 and prohibitive α = 0.88), *loyalty/supervisor commitment* (α = 0.93), and *neglect/withdrawal* (α = 0.94).

### Preliminary results: comparison between studies

First, we looked at the means and standard deviations of Study 1, 2, and 3 to see if our studies are comparable in terms of sample characteristics relevant to this study. Negative affectivity, hostile attribution style, anxiety (Study 2 and 3), and irritation were comparable between Study 1, 2, and Study 3, indicating that the samples are equivalent in terms of participants' characteristics. However, perceived abusive supervision was clearly lower in Study 3 (*M* = 1.62, *SD* = 0.85) than in Study 1 (*M* = 2.82, *SD* = 1.11) and Study 2 (*M* = 2.60, *SD* = 1.14), reflecting that in our experimental studies, half of our sample was confronted with abusive supervision whereas the phenomenon is rare in the field study, comparable to previous studies. We also found higher levels of negative reactions in Study 2 compared to Study 3, reflecting the manipulation of abusive supervision. It is also noticeable that stable characteristics (apart from hostile attribution style) and irritation were more strongly related to the perception of abusive supervision in the field Study 3 than in Study 1 and 2, reflecting the ambiguity of actual leader-follower interactions.

All our follower characteristics, that is, irritation (*r* = 0.42), anxiety (*r* = 0.28), hostile attribution style (*r* = 0.30), and negative affectivity (*r* = 0.51) were positively and significantly related to perception of abusive supervision perceptions (see Table 6). However, in a multiple regression analyses, only irritation, anxiety, and negative affectivity remained significant predictors of abusive supervision perceptions, and were thus taken forward as control variables.

**Table 6:** Means, standard deviations, and intercorrelation (Study 3).

	**M**	**SD**	**1**	**2**	**3**	**4**	**5**	**6**	**7**	**8**	**9**	**10**	**11**
Abusive supervision	1.62	0.85											
Irritation	2.70	0.87	0.42^**^										
Anxiety	3.12	1.02	0.28^**^	0.65^**^									
Negative affectivity	1.83	0.81	0.51^**^	0.64^**^	0.63^**^								
Hostile attribution style	2.81	0.77	0.30^**^	0.36^**^	0.31^**^	0.40^**^							
Attribution supervisor(r)	3.82	1.78	−0.22^**^	−0.08	−0.13^*^	−0.23^**^	−0.25^**^						
Intentionality	3.09	1.19	0.33^**^	0.13^*^	0.05	0.14^*^	0.08	−0.08					
Turnover intention	2.73	1.45	0.40^**^	0.36^**^	0.17^**^	0.32^**^	0.13^*^	0.14^*^	0.22^**^				
Supervisor commitment	2.94	1.12	−0.44^**^	−0.19^**^	−0.07	−0.13^*^	−0.10	0.00	−0.20^**^	−0.42^**^			
Prohibitive Voice	3.14	0.92	0.08	0.02	−0.11	−0.05	−0.02	−0.07	0.17^**^	−0.03	0.11		
Promotive Voice	3.54	1.01	−0.10	−0.10	−0.15^**^	−0.15^**^	−0.07	0.04	0.06	−0.17^**^	0.24	0.56^**^	
Withdrawal	2.12	1.25	0.65^**^	0.34^**^	0.18^**^	0.33^**^	0.26^**^	0.23^**^	0.22^**^	0.44^**^	−0.55^**^	−0.04	−0.10

* p < 0.05 (2-tailed)

** *p < 0.01 (2-tailed)*.

### Results

Perceived abusive supervision was positively related to attribution to the supervisor's control (*r* = 0.22) and to intentionality (*r* = −0.33). Perceptions of abusive supervision were related to outcomes as expected: Turnover intention (*r* = 0.40), supervisor commitment (*r* = −0.44), and withdrawal (*r* = 0.65) but not to prohibitive voice (*r* = 0.08) or promotive voice (*r* = −0.10), lending partial support to H1.

Before conducting moderated regression analyses, we centered perceived abusive supervision and the moderators (intentionality, and attribution to the supervisor) and then calculated the interaction terms. As depicted in Table 7, none of the interactions between abusive supervision and attribution of intentionality became significant, thus H3 was not supported for attribution to intentionality.

**Table 7:** Moderated regression analyses attribution to intentionality and reactions (Study 3).

	**Turnover intention**			**Supervisor commitment**			**Withdrawal**			**Prohibitive Voice**			**Promotive Voice**		
	**B**	**beta**	**Δ *R*^2^**	**B**	**beta**	**Δ *R*^2^**	**B**	**beta**	**Δ *R*^2^**	**B**	**beta**	**Δ *R*^2^**	**B**	**beta**	**Δ *R*^2^**
Model 1			0.10^**^			0.02^*^			0.11^**^			0.00			0.02^**^
Constant	1.68			3.28			1.19	0.33		3.24			3.89		
Negative affectivity	0.57	0.32^**^		−0.18	−0.13^*^		0.51			−0.06	−0.051		−0.19	−0.152	
Model 2			0.09^**^			0.19^**^			0.31^**^			0.04^**^			0.01
Constant	2.21			2.64			2.13			3.38			3.84		
Negative affectivity	0.28	0.16^**^		0.167	0.12^*^		−0.00	−0.00		−0.13	−0.12		−0.16	−0.13	
Abusive supervision	0.48	0.28^**^		−0.63	−0.48^**^		0.94	0.64		0.09	0.09		−0.08	−0.07	
Intentionality	0.13	0.11^*^		−0.06	−0.06		0.02	0.02		0.13	0.16^**^		0.08	0.10	
Model 3			0.00			0.00			0.01			0.00			0.00
Constant	2.19			2.61			2.17			3.37			3.85		
Negative affectivity	0.29	0.16^**^		0.17	0.12^*^		−0.01	−0.01		−0.13	−0.12		−0.17	−0.13^*^	
Abusive supervision	0.45	0.27^**^		−0.67	−0.51^**^		1.01	0.69		0.06	0.06		−0.06	−0.05	
Intentionality	0.14	0.12^*^		−0.05	−0.05		0.00	0.00		0.13	0.17^**^		0.08	0.09	
Interaction AS × Intentionality	0.05	0.03		0.07	0.07		−0.10	−0.08		0.04	0.04		−0.03	−0.03	

* p < 0.05 (2-tailed)

** *p < 0.01 (2-tailed)*.

The results for the moderation effect of attribution to the supervisor's control on the relationship between perceived abusive supervision and reactions showed some significant results: For both aspects of voice as well as for supervisor commitment, the interactions became significant (see Table 8).

**Table 8:** Moderated regression analyses attribution to supervisor and reactions (Study 3).

	**Turnover intention**			**Supervisor commitment**			**Withdrawal**			**Prohibitive voice**			**Promotive Voice**		
	**B**	**beta**	**Δ *R*^2^**	**B**	**beta**	**Δ *R*^2^**	**B**	**beta**	**Δ *R*^2^**	**B**	**beta**	**Δ *R*^2^**	**B**	**beta**	**Δ *R*^2^**
Model 1			0.10^**^			0.02^*^			0.11^**^			0.00			0.02^**^
Constant	1.68			3.28			1.19			3.24			3.89		
Negative affectivity	0.57	0.32^**^		−0.18	−0.13^*^		0.51	0.33^**^		−0.06	−0.05		−0.19	−0.15	
Model 2			0.08^**^			0.19^**^			0.32^**^			0.02			
Constant	2.24			2.67			2.17			3.41			3.85		0.00
Negative affectivity	0.27	0.15^*^		0.15	0.11		−0.03	−0.02		−0.15	−0.13^*^		−0.17	−0.13^*^	
Abusive supervision	0.54	0.32^**^		−0.67	−0.51^**^		0.93	0.64^**^		0.14	0.13		−0.04	−0.04	
Attribution supervisor	0.03	0.04		0.06	0.09		0.07	0.09^*^		0.04	0.07		0.00	0.00	
Model 3			0.00			0.03^**^			0.00			0.02^*^			0.03^**^
Constant	2.23			2.69			2.17			3.43			3.88		
Negative affectivity	0.28	0.16^*^		0.12	0.08		−0.03	−0.02		−0.17	−0.15^*^		−0.20	−0.16^*^	
Abusive supervision	0.54	0.32^**^		−0.67	−0.51^**^		0.93	0.64^**^		0.14	0.13		−0.05	−0.04	
Attribution supervisor	0.05	0.06		0.03	0.05		0.06	0.09^*^		0.02	0.04		−0.02	−0.04	
Interaction AS × Attribution supervisor	-0.05	−0.07		0.11	0.19^**^		0.00	0.00		0.07	0.14^*^		0.09	0.18^**^	

* p < 0.05 (2-tailed)

** *p < 0.01 (2-tailed)*.

**Figure 8:**
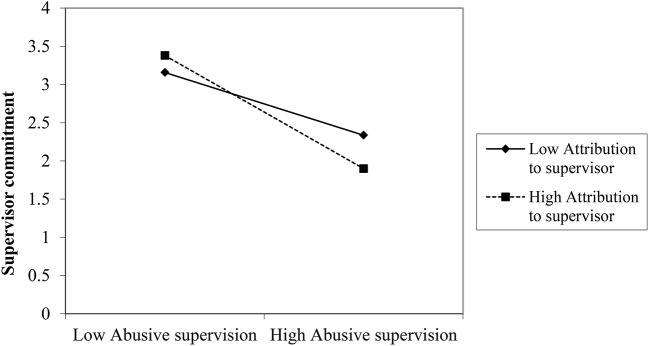
Interaction between abusive supervision and attribution to the supervisor on supervisor commitment (Study 3).

**Figure 9:**
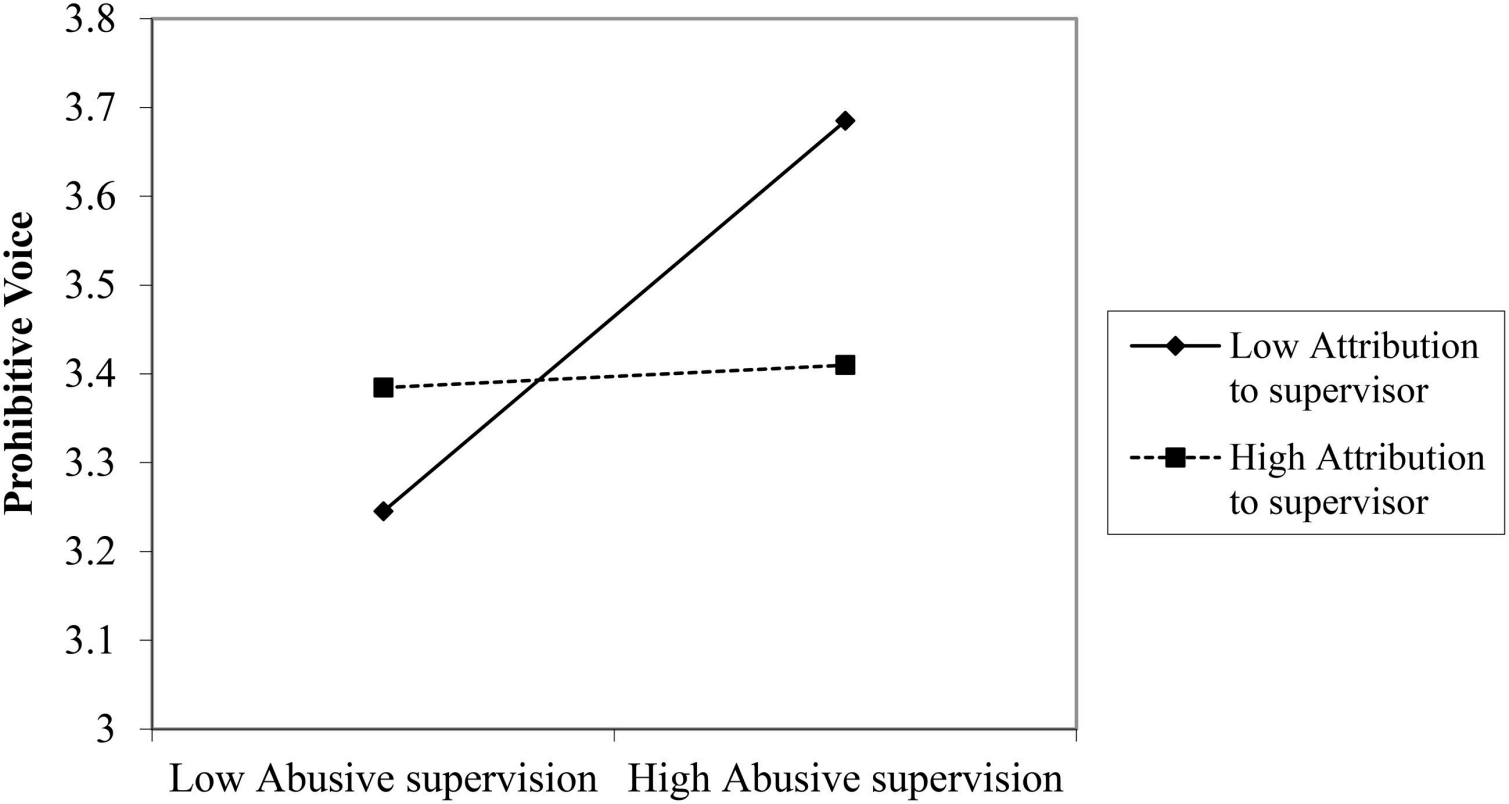
Interaction between abusive supervision and attribution to the supervisor on prohibitive voice (Study 3).

**Figure 10:**
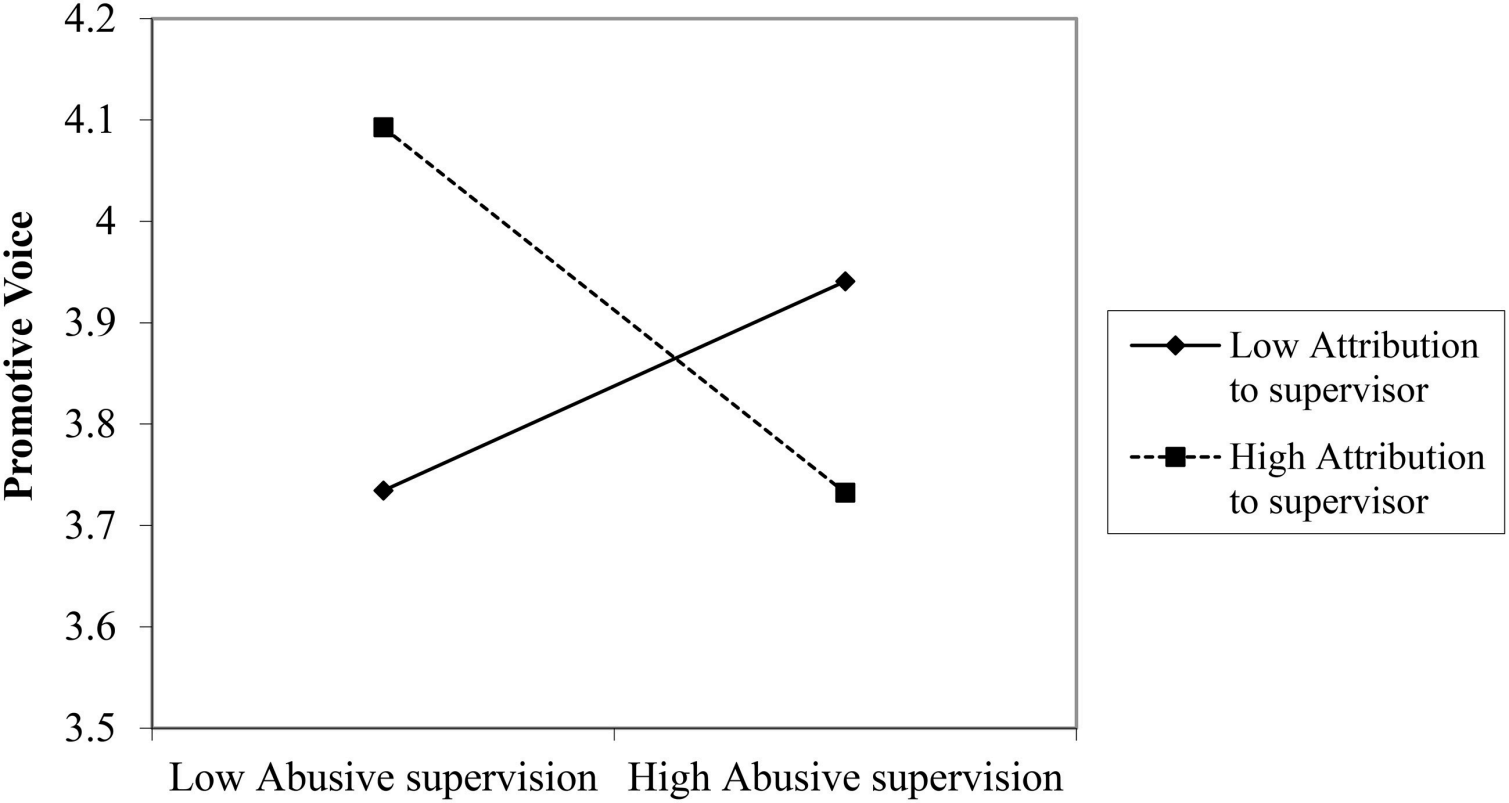
Interaction between abusive supervision and attribution to the supervisor on promotive voice.

We plotted the interactions to further examine the moderation effects. Figures 8–10 depict the interaction effects. The relationship between perceived abusive supervision and supervisor commitment was negative for both high and low attribution pf control to the supervisor but the relationship was stronger for high attribution, indicating that attribution makes the negative effect of perceived abusive supervision on supervisor commitment stronger.

The relationship between perceived abusive supervision and prohibitive voice was almost zero for high attribution but positive for low attribution, meaning that participants perceiving high abusive supervision but who do not attribute this behavior to their supervisor feel less inhibited to speak up. A similar pattern emerges for promotive voice: Here the relationship between perceived abusive supervision and promotive voice was also positive for low attribution but it was negative for high attribution, indicating that participants perceiving high abusive supervision and hold their supervisor responsible for his/her behavior, are less likely to be proactive in their voice behavior. Thus, the results lend support to H3 for attribution to the supervisor's control.

## General discussion

The aim of the study was to deepen our understanding of destructive leadership, especially with regard to how followers react to abusive supervisor behaviors and which role perceptions and attributions play in this process. Using an experimental approach, we examined in how far followers react differently to different levels of abusive leadership and how this relationship is mediated by perceptions of abusive leadership, controlling for individual variables which have been previously identified as causing rater biases (e.g., Hansbrough et al., 2015; Brees et al., 2016). It was expected that the effects of differences in behavior were stronger in the experimental setting whereas subjective biases should be stronger in the field context, where behavior is less clear and more ambiguous. Experimental vignette designs complement field research as they enhance experimental realism and also allow researchers to manipulate and control independent variables, thereby simultaneously enhancing both internal and external validity (Aguinis and Bradley, 2014). Moreover, we wanted to clarify if perceivers are able to distinguish between different levels of leadership behavior in order to provide evidence for the validity of different concepts of negative leadership behavior (Schilling, 2009; Schyns and Schilling, 2013).

We report results of overall four studies: one pre-study, two experimental studies, and one field study. Our first three studies used vignettes of leader behavior. In all those studies, we found the expected differences in follower perceptions (though not all significant) between different levels of abusive leader behavior from not at all abusive (constructive) to strong abusive. Hence, we can conclude that actual leader behavior is a strong predictor of perceived abusive supervision. However, in the pre-study and in Study 2 those differences were not significant between laissez-faire and mild abuse. There is a discussion in the literature that laissez-faire leadership is perceived as abusive as abusive supervision (Skogstad et al., 2014). In our study, we directly compared the perceptions of laissez-faire to different levels of abusive supervision, and our results indicate that, when leader behavior is systematically varied, the perceptions of abusive supervision are comparable for laissez-faire and mild abusive supervision, also in terms of liking and generalized leadership impressions. Hence, our results indicate that laissez-faire is comparable to mild abusive behavior but that strong abusive behavior is worse in terms of leadership perceptions.

In Study 1, we included reactions toward abusive supervisor behavior. Here we found the expected differences depending on the described leader behavior. Constructive leadership always elicited the most positive reaction; laissez-faire and mild abuse were comparable in terms of reactions. For quite, anger, and accept, strong abuse also did not differ from laissez-faire and mild abuse. All the negative forms of leadership seem unacceptable to our participants. As expected, we found that perceptions of abusive supervision are related to those reactions. However our mediation analysis showed that perception does not capture the entire effect of behavior and some follower characteristics (irritation, hostile attribution style, and negative affectivity) are related to followers' reactions. This also means that follower reactions cannot be simply reduced to sensitivities of the followers. Instead leader behavior is a crucial factor in reactions toward negative leader behavior. The measurements for reactions were improved in Study 2. Here, we found again that most reactions differed as expected depending on the described leader behavior (apart from the effect for prohibitive voice). However, looking at the mean differences for the vignettes, again, laissez-faire did not differ from mild abuse. Overall, it seems that laissez-faire and mild abuse are perceived similarly and provoke similar reactions. This is interesting and adds to the discussion of how abusive laissez-faire leadership is perceived (Schyns and Schilling, 2013; Skogstad et al., 2014). Schyns and Schilling (2013) argue that there is a clear qualitative difference between non-leadership and active supervisor hostility. However, it seems that laissez-faire leadership in terms of the perception and reactions of followers is more negative than this conceptual distinction may lead us to expect.

Our results may point to an explanation for the result that laissez-faire and abusive supervision show similar relationships with important variables in the workplace (cp. Skogstad et al., 2014). It seems safe to assume—like in our own field study—that abusive supervision in real work settings will mainly come in mild forms which seems to provoke reactions and consequences which are similar to laissez-faire. Strong abusive supervision (as presented in one of our vignettes) is likely a rather seldom phenomenon so that field studies will find it difficult to capture its consequences.

The correlations between perceptions of abusive supervision and reactions were all significant, again apart from prohibitive voice. The latter result was similar in Study 3 (field study), where perception of abusive supervision was related to reactions apart from the two aspects of voice. Overall, our results replicate previous studies showing that abusive supervision is related to negative outcomes such a lower supervisor commitment and higher turnover/withdrawal from the supervisor (Martinko et al., 2013; Schyns and Schilling, 2013; Zhang and Liao, 2015). Even more, in our studies, due to the experimental designs, we could show that this effect is based on actual leader behavior.

As mentioned before, we examined and confirmed most of the postulated mediating effects of the perception of abusive supervision on the relationship between leader behavior and reactions. In Study 1, we found both direct and indirect effects of leader behavior on follower reactions, apart from anger where the indirect effect was not significant. We could replicate the same pattern of results for the mediation analyses in Study 2, apart from the effect for prohibitive voice. We thus found that abusive leader behavior is related to outcomes and that this relationship is partly mediated by perceptions of abusive supervision, emphasizing that both behavior and perception are relevant for reactions to outcomes which indicates evidence for the importance of this distinction (Martinko et al., 2013).

In terms of the reactions, it seems that voice is ambivalent in relation to abusive supervision. It likely contains two aspects that are differently influenced by abusive supervision, namely, complain (e.g., about the abuse) in study 1 and speaking up in study 2. While followers of abusive supervisors complain more, for example to higher authorities, they are less likely to speak up to the supervisor, as they lack of trust and may fear negative consequences. This also is reflected in our partly different results for promotive and prohibitive voice. In both Study 2 and 3, abusive supervision was not related to prohibitive voice, which is related to pointing out problems in the workplace. As abusive supervision is likely to be part of the problem, different effects could occur: Some employees might speak out against abusive supervision to address the issue while most feel hindered to do so due to fear of retaliation from the supervisor (cp. Schyns and Schilling, 2013). In Study 1, participants said that they would complain more under abusive supervision but in Study 2 and 3, voice was negatively (promotive, Study 2) or unrelated to abusive supervision (Study 3). These mixed results are in line with prior research which found interactions between interactions between person-centered and situational factors play an important role in predicting voice behavior (e.g., LePine and Van Dyne, 1998; Wei et al., 2015). Future research should try to disentangle who the voice is addressed to (the supervisor, peers, or higher up leaders or HR) and what the voice is about (the supervisor vs. other aspects of work) to further clarify these relationships.

We also investigated the role of attributions in this mediating relationship (Study 2). Specifically, we investigated if and to what degree attributions moderated the relationship between the perception of abusive leadership and reactions (Study 2 and 3). In Study 2, we found that attribution to intentionality moderated the relationship between perception of abusive supervision and reactions (Study 2). Here attribution of intentionality reduced the relationship between perception of abusive supervision and reactions, in so far that intention played no role in reactions for strong abusive supervision but it strengthened negative reactions when milder abuse was perceived. This is interesting as it shows that once abusive supervision is strong, it does not seem to matter in how far that behavior is shown intentionally; the reactions remain strong. However, where abuse is milder (and, thus, likely more ambiguous to interpret), the way perceivers attribute the behavior is more relevant in determining their reactions (cp. Liu et al., 2012). This is also potentially relevant in the field context, where behavior is likely to be more ambiguous and very strong abuse is (thankfully) rather a rare phenomenon.

Interestingly, attribution to intentionality was unrelated to abusive supervision in Study 2 but related to the perception of abusive supervision in the field Study 3, likely to reflect ongoing dynamics in the relationships between followers and supervisors in the field. However, intentionality was related to most outcomes in both studies, indicating that attributions can influence outcomes directly (cp. Martinko et al., 2013).

We further investigated the moderating role of attributions in Study 3. In addition to attribution of intentionality, we also included attribution to control of the supervisor. We could not replicate the results of Study 2 regarding the moderating effect of attribution of intentionality in our field Study 3. This might be the case because we did not differentiate between types of intention. So, for example, followers might perceive that a leader is intentionally abusive to achieve a certain goal or intentionally abusive to hurt followers. Tepper (2007) differentiates two types of attributions for abusive supervision, namely, harm of others or achieving an objective (e.g., performance). Liu et al. (2012) argue that attribution to performance promotion motives moderate the relationship between abusive supervision and creativity in a different way from attribution to injury initiating motives. Indeed, both attributions were differently related to the perception of abusive supervision. Liu et al. also found the suggested different moderation effects: While the relationship between abusive supervision and creativity was negative for both high and low performance attribution, it was most negative for low performance attribution. For injury attribution, the effect was higher for high injury attribution. Thus, future research should differentiate between performance and injury attribution to examine in how far they differentially influence on the relationship between abusive supervision and reactions.

While our results for the attribution of intentionality failed to become significant, we found moderating effects of attribution to control of the supervisor in Study 3. For strong attribution to the supervisor the relationship between abusive supervision and reactions was stronger than for weaker attributions. Thus, participants who did not hold their supervisor responsible for his/her abusive behavior showed more voice and were slightly more committed to this supervisor. Here, it would be interesting for future research to differentiate further what other attributions might be relevant. For example, we would assume that the effects of abusive supervision on reactions might be mitigated by attributions toward circumstances (external and unstable attributions: e.g., leader stress, time or task pressure; cp. Weiner, 1986; Martinko et al., 2011). It would also be interesting to examine in how far such excuses would hold up over time. That is, even when abusive supervision is attributed toward circumstances outside the control of the supervisor, followers might eventually still react more negatively as the power of the excuse runs out.

We found some interesting results with respect to the discussion around the effects of follower characteristics on the perception of abusive supervision (e.g., Martinko et al., 2013; Brees et al., 2016). In Study 1, we found no effect of participant characteristics on the perception of abusive supervision, contrary to our expectations based on the literature regarding perception biases. However, this shows clear differences in leader behavior displayed in the descriptions as they leave little room for rater biases. In Study 2, there was a slight effect of negative affectivity effect, but the strongest effect emerged in Study 3, lending support to the assumption that actual leader behavior leaves more room for interpretation based on perceiver characteristics than our described supervisors. This also means that when leaders behave unambiguously, perception effects are likely a lot lower than when behavior is ambiguous. In that sense, our vignettes can be described as strong situations according to Mischel (1977), meaning that fewer effects of personality on perception can be expected.

We also contribute to our understanding of the influence of follower characteristics on the perception of abusive supervision vs. on the behavior of abusive supervisors. For example, Wang et al. (2015) argue that supervisors might treat followers high in neuroticism in an abusive way based on the victim precipitation approach. Brees et al. (2016), in contrast, argue for perception effects. Due to the experimental design of our first two studies, we can show that where behavior is clearly positive or negative, few rater effects occur. Future research needs to further disentangle the relationship between follower characteristics and abusive supervision. Specifically, it would be interesting to study circular effects over time where follower characteristics influence leader behavior which then lead to changes in follower characteristics over the period of abuse.

## Limitations

Experimental studies are often criticized for their lack of external validity (Aguinis and Bradley, 2014). However, in order to show causal effects, experiments are invaluable. In terms of the moderating effects of attribution, we conducted a field study to examine in how far our results replicate. One strength of our studies reported here is that the differences in the means and standard deviations between Study 2 and Study 3 lend support to the validity of our measurement as well as our manipulation of abusive supervision. However, the correlations were not affected by those mean differences (bottom or ceiling effects). In addition, in all three experimental studies, the mean values for abusive supervisions and differences between the vignettes were comparable, indicating the validity of our manipulations. We also found no differences in terms of the participants' characteristics in the three studies but found the expected differences between the experimental and field studies in terms of perceptions of abusive supervision and reactions. This lends support to our approach of using experimental studies and combining them with a field study.

In our experimental studies, we asked our participants to anticipate how they would react to the leader behavior. Again, this could be criticized as being artificial. However, our results were mainly replicated in the field study, showing that we can draw conclusions from experimental studies to the field. Indeed, one might argue that the fact that our participants showed reactions after a limited exposure to leader behaviors constitutes a more conservative test of reactions toward abusive supervision. The exception here was voice where it seems that participants in the experimental studies might over-estimate their engagement in voice when exposed to abusive supervision. What could be interesting for future research is to assess physiological stress measures after the exposure to abusive supervision in an experiment. This would go beyond anticipated reactions as we investigated them here.

While we pre-tested our vignettes, we only used abusive supervision, liking, and leadership impressions as manipulation checks. Ideally, we would have included a measurement for constructive and, more importantly, laissez-faire leadership. The latter would have been useful to check in more detail in how far laissez-faire and mild abusive differ from each other.

Our studies employed the description of only one situation. This is a clear limitation as results relating to a variety of situations would have lend more confidence to our results. However, our vignette is quite detailed and describes a typical leader follower interaction. Future studies could employ a different situation to add confidence to the generalizability of our results across situations. However, they have to take into account either the issue of a larger sample size (between participant design) or risk participant fatigue (within participant design).

Future research might also consider using videos instead of written vignettes. While they are easier to present, written vignettes are likely to be seen as less realistic than videos. However, when constructing videos, researchers have to be careful not to vary appearance as faces of leaders already lead to leadership impressions (Antonakis and Dalgas, 2009; Trichas et al., 2017). They also have to carefully manipulate tone of voice or facial expressions to best express the different leadership styles.

## Practical implications

In terms of practical implications, we can conclude that leader behavior is important and that negative leader behavior needs to be addressed by organizations. At the same time, leaders should be made aware that in practice, their behavior might come across as ambivalent and could be subject to rater effects and thereby lead to negative effects in their followers. For example, we found that laissez-faire is perceived as similar and reacted to in a similar as mild abusive supervision. This points to the usefulness of integrating negative behaviors, such as abusive supervision, into 360 degree feedback and to carefully disentangle interactions both for the benefit of the leader and the follower.

## Conclusion

The aim of this study was to examine the differentiated effects of abusive leader behavior on follower reaction as mediated by the perception of abusive supervision. According to our results both are important and, similar to constructive leadership, there are effects of raters on the perception of abusive supervision, specifically in the field where behavior differences may be less clear than in an experiment. Perception of abusive supervision mediates the relationship between leader behavior and reactions, lending support to the relevance of perceptions in leadership research and the necessity to take into account perception effects when assessing leadership and its outcomes. We also found that attributions can influence the strength of reactions, lending support to the notion that some people suffer more under abusive supervisors than others.

## Author contributions

All authors contributed to the design of the studies. BS did the majority of the writing up and the analyses, which JF supported and checked. JS was involved in all the writing and thinking process.

### Conflict of interest statement

The authors declare that the research was conducted in the absence of any commercial or financial relationships that could be construed as a potential conflict of interest.

## Footnotes

^1^Since all data reported here were collected using panel providers, no informed consent forms were used. Participants in panel studies can chose to take part in studies for a small recompensation. The first page of each study contained information about the study as well as that data would be treated confidentially and used for scientific purposes. Ethical consent from the first author's institutions was gained via self-assessment as no particular ethical issues were detected that would merit the involvement of an ethics committee. This is standard practice at both institutions.

^2^We conducted the same manipulation check in Study 1 with comparable results (see Table 1). The reliability for liking was α = 0.97. For Generalized Leadership Impression, the reliability was α = 0.91.

^3^Testing the model without control variables does not change the results.

^4^In order to plot the interaction, we conducted moderated regressions using the vignettes as a control variable.
