# Supplementary material for: Is It Me or You?—How Reactions to Abusive Supervision Are Shaped by Leader Behavior and Follower Perceptions
Source: Front Psychol. 2018 Jul 27;9:1309. doi: 10.3389/fpsyg.2018.01309 (PMC6073698; doi:10.3389/fpsyg.2018.01309)
Supplement: Supplementary file 1 [file Data_Sheet_1.docx]

**Appendix**

*Constructive leadership vignette*

Thank you for coming straight away. I asked you to come here to talk about the presentation for the project tomorrow. As you know time is pressing. You are aware that the session for the reorganisation project is set for tomorrow and that we have to present our suggestions. You know that all in all I value your work quite highly. This time, however, I’ve to say that I’m not satisfied with your preparation for the presentation. We can’t show up tomorrow at the session with this presentation. But maybe you’ve important reasons for the delay that I am not aware of. It doesn’t matter, it’s important to me that we discuss the situation openly and honestly, and above all that we find a solution.

Let me briefly outline my point of view and after that I’d like to hear your opinion. As you remember, we fixed the deadline for the presentation to be two days ago so that we would have enough time to make changes and additions and deal with any potential remaining issues. I did ask you if you could manage or if you needed support. If I understand correctly, the presentation is just half finished and we have the session tomorrow. Now, it’ll be a tight squeeze for the simple reason that we lack time. What I’ve seen of the presentation so far is – I’m afraid to say – not sufficient at all.

In my view some aspects are already quite all right. The background to the problem is well demonstrated and illustrated well with figures. However, the wording for the project goals is not precise enough and will make a later evaluation of success very difficult. We need a clear structured goal catalogue here, ideally including intermediate goals as well. Especially the cost saving we aim at has to be stated precisely and should be demonstrated on the basis of each particular item. Concerning the actions to be taken, I noticed that your proposals are appropriate in principle and I really like some of the ideas, but the reasoning is missing and we still need concrete examples. Otherwise no one will be able to follow us and no one will buy into our concept. As for the schedule and the resources, they look equally vague; this definitely needs quite a few changes.

I’m afraid we are in an unfortunate situation if the presentation tomorrow doesn’t go really well. This must not happen. If necessary, I will jump in this time and support you as far as my resources permit it. However, I would like you to make constructive suggestions on how to save the situation. Based on your expertise I trust that you will manage to do so.”

Suddenly the telephone is ringing, and your supervisor turns away from you to pick up. “Ah Joe, hello. Do you mind if I call you back? I am in an important conversation; we’ve an urgent deadline that I need to take care of. I am sure it won’t take long, so I’ll call you back soon. ... Thanks. Talk to you soon.” Your supervisor hangs up and turns back to you and continues: “Where were we?”

“Indeed, yes. The situation can’t be changed now. When this issue is over we’ll have to talk about the reasons why the scheduling got out of hand. I don’t want us to be in such an awkward situation again. For tomorrow, there’s nothing we can do but catch up on what is missing. I urgently request that you present me with the finished presentation at 9.00 o’clock tomorrow. For this, I need your expertise. I’m sure that we’ll find a solution. Please, fetch your paperwork so that we can have a look together and are able to coordinate the critical issues.”

As you leave the office you stop outside to take a deep breath. Through the half open door you hear your supervisor saying on the phone. “Yes, Joe? Listen, it’ll take more time here after all. I will call you later, when I’ve finished this, all right?”

*Laissez-faire leadership vignette*

“You asked for this meeting to talk about the presentation for the project tomorrow. To come straight to the point, I hardly have time to look at it and so I could only skim through your presentation. You know that I’m very busy just now working on other issues, really extremely occupied. That is why I see you as in charge. So I don’t want to get too involved. That’s how it was supposed to be. I can’t take care of things like this myself. Now, I don’t know what to make of what you provided me with, but this isn’t quite finished yet, is it?

I don’t have the presentation right here but as far as I remember it wasn’t quite perfect yet, was it? We did not agree a date or anything, but I assumed that you would start on time so that the project would be running smoothly. Were you able to discuss any changes, additions, or open questions with your colleagues? Now I’m not sure that that is still in option until tomorrow, what do you think? I’m obviously worried there will be a problem and I can really do about this as my agenda is full.

Well, at this moment, I’m involved in so many projects that I really have to rely on staff working autonomously. That is very important. As the boss, it must be possible for me to stay out of such operational aspects of the business, so that I can focus on my own concerns in this organisation. At present, I am really busy planning the department day out. You have no idea about how much work is entailed. I forwarded you the project documents and it was self-explanatory what had to be done by when. I mean, we are all professionals and hence, I hope, we don’t need to talk everything through over and over again? If need be, talk to colleagues and get feedback from them. I am sure they can comment better than I can. You really need to do this autonomously and I think asking your colleagues shouldn’t be an issue, should it?

Of course, you would really put yourself in an awkward situation if your presentation tomorrow runs badly. I hope for the best. Well, what more can I tell you now? Take care of this, good luck, maybe it will still work, difficult to say. Obviously, I want to avoid having the whole department appears in a bad light. That would be most awkward for all of us. Well, I think now...”

Suddenly the telephone is ringing, and your supervisor turns away from you to pick up the phone. “Ah Joe, hello, thanks for calling. Do you mind if I call you back? I am in a conversation with an employee at the moment, some things we need to clarify. ... No, it’s not that important, but we needed a brief conversation. I am sure it won’t take much longer. Thanks. Talk to you soon.” Your supervisor hangs up and turns back to you and continues: “Where were we?”

“Indeed, yes. I can’t do anything about this situation now. When the presentation is finished, you should think about how things like this can be avoided in future. I really don’t want you to be in the same situation again. But for now you have no choice but to play catch up. See that you finish this on time and that you can deliver a finished presentation. Now I’ve got to tend to other important matters. There are several burning issues. Maybe it is necessary to put in a nightshift. Well then, best for you to get back to work.”

As you leave the office you stop outside to take a deep breath. Through the half open door you hear your supervisor saying on the phone. “... No, no, nothing of importance. Let us get back to our issue: Please tell me about the results of the meeting with the board.”

*Mild abusive leadership vignette*

“I demanded that you come to my office to talk about the presentation tomorrow; and it’s obviously high time. To come straight to the point, and I’ll put this bluntly: I’m really dissatisfied with your performance; it’s not acceptable. I can’t follow what you were thinking... What on earth were you thinking? You have been working on this intensely, haven’t you? What you have delivered here won’t work, and there is no way to sugar-coat this matter. I thought the presentation was running smoothly and now I look stupid seeing your output, I was really shocked.

We clearly agreed that the presentation was due two days ago so that there would be enough time to make changes and additions, and to resolve any remaining issues. We had discussed this very clearly. That won’t be possible anymore, because we have run out of time. In addition, what I’ve seen of the presentation so far does not suffice at all. Obviously, you started way too late or simply did not put in the necessary diligence.

I know that we are all very busy at the moment, but this can’t be an excuse for such a performance. Apart from that, you had clear and precise instructions – there is nothing to argue. That obviously didn’t work. I surely am not expecting too much of you to finish my assignments on time. I need to be able to rely on you. And unfortunately that wasn’t always the case in the past either, as you will remember, but I don’t want to go back there now. Taking into account the problems you had with your colleague Alex recently, that doesn’t reflect very well on you, I have to say.

Now we’re in a really awkward situation. If this is presented badly tomorrow afternoon you also got me into trouble. But let me tell you, it’s not acceptable for me. I’ve worked hard for my current position and my reputation here. I won’t let it be damaged in this way. You and me, we know who is responsible for this and if necessary that will be made clear to everyone else.”

Suddenly the telephone is ringing, and your supervisor turns away from you to pick up the phone. “Ah Joe, hello. Do you mind if I call you back? I am in an important conversation with a co-worker; something is going wrong again and, obviously, I’ve to deal with this to avert disaster. I am sure it won’t take long… Thanks. ... Talk to you soon.” Your supervisor hangs up and turns back to you and continues: “Where were we?”

“Indeed, yes. The situation can’t be changed. When this thing is over we’ll need to have a serious conversation about this. I don’t want you to put me in such a situation again. For tomorrow, there’s nothing else to do than for you to urgently catch up. I expect you to present me with the finished presentation at 9.00 o’clock – I don’t care how you achieve this, and if you’ve to work a night-shift, you brought it all on you yourself. I can only hope that you understood me, as I am really serious. I won’t accept it, let me repeat this, will not accept it, if there’s no finished presentation on my desk by 9 o’clock tomorrow. We don’t need to talk about what’s going to happen otherwise, do we? So, here we go, get back to work!”

As you leave the office you stop outside to take a deep breath. Through the half open door you hear your supervisor saying on the phone. “...Hopefully, I made clear what’s not acceptable here. I hope that it has finally hit home.”

*Strong abusive leadership vignette*

“Why do you think I’ve summoned you? Well? No idea? I wish I had your nerves, but maybe it’s just your stupidity or ignorance, probably both. What am I supposed to do with you? Maybe you can tell me that? The situation is more than annoying, it’s ridiculous. I couldn’t believe my eyes when I saw how far along you are with the presentation. I was truly shocked. In short, a catastrophe, an absolute disaster; the liberties you take. No way! I could have hired anyone from the street and that person would likely have done a better job. In short, it’s outrageous and an impertinence, and there’s no way to sugar-coat this matter.

We’d unambiguously agreed that the presentation should be ready two days ago. A deadline is a deadline, for heaven’s sake! Do you even listen to me when I discuss things with you? How shall we get this all done now? Obviously you started way too late and hadn’t grasped at all what it’s about. But that’s not first time for you. By now, I’m starting to seriously wonder how you managed to keep your job for so long and who conceals your inaptitude.

And please don’t start with any excuses or apologies – preferably blaming someone else – that’s just ridiculous! We are all very busy. You had clear and precise instructions – but you failed to follow them. It borders on refusal to work and sabotage. You are the problem! Remember what else you’ve messed up over time, for example, the accounting error last week? And I covered your back that time. Who knows what will be uncovered if somebody takes a closer look. By the way, some colleagues complained about you and apart from that I’ve been told some more things – but that’s not the issue today.

Now we’re in an awkward situation, most of all you got me into trouble, if this is presented badly tomorrow afternoon. But I promise you, I won’t let you destroy my reputation. You’ll answer for this personally. You will go down, and you’ll go down alone! I’m glad that I recorded the briefing where I gave you clear instruction, so there’s nothing to deny – in case it was your plan to do so.

Suddenly the telephone is ringing, and your supervisor turns away from you to pick up the phone. “Ah Joe, hello. Do you mind if I call you back? We’ve problems again with you know who... Yes, sitting right in front of me... A catastrophe... I would really like to know who is hiring people like that. Can you tell me, what I am supposed to do? I’m probably too soft-hearted... It won’t take long now... Talk to you soon.” Your supervisor hangs up and turns back to you and continues: “Where were we?”

“Indeed, yes. The situation can’t be changed now anyway. When this is over we’ll have to have serious words. You’ll never again put me in such a situation, is that clear? For tomorrow there’s nothing else to do than for you to sort out this mess. Tomorrow at precisely 9 o’clock, the presentation will be finished – I don’t care at all how you achieve it. Pull a night-shift, do whatever is necessary, I don’t have much hope for you anyhow. I can hardly rely on you understanding me considering your intellectual capabilities, but I’m dead serious and I won’t accept it, not on any terms, if there isn’t a finished presentation by 9 o’clock tomorrow. Otherwise... Well, you can work that out for yourself. So remember for once that you’re here to work...”

As you leave the office you stop outside to take a deep breath. Through the half open door you hear your supervisor saying on the phone. “There, I have raised the pressure and I made up some things, but some people just need it this way to get it.”
